# Structural trends in the dehydrogenation selectivity of palladium alloys†

**DOI:** 10.1039/d0sc00875c

**Published:** 2020-05-05

**Authors:** Stephen C. Purdy, Ranga Rohit Seemakurthi, Garrett M. Mitchell, Mark Davidson, Brooke A. Lauderback, Siddharth Deshpande, Zhenwei Wu, Evan C. Wegener, Jeffrey Greeley, Jeffrey T. Miller

**Affiliations:** Davidson School of Chemical Engineering, Purdue University West Lafayette IN 47907 USA jeffrey-t-miller@purdue.edu jgreeley@purdue.edu

## Abstract

Alloying is well-known to improve the dehydrogenation selectivity of pure metals, but there remains considerable debate about the structural and electronic features of alloy surfaces that give rise to this behavior. To provide molecular-level insights into these effects, a series of Pd intermetallic alloy catalysts with Zn, Ga, In, Fe and Mn promoter elements was synthesized, and the structures were determined using *in situ* X-ray absorption spectroscopy (XAS) and synchrotron X-ray diffraction (XRD). The alloys all showed propane dehydrogenation turnover rates 5–8 times higher than monometallic Pd and selectivity to propylene of over 90%. Moreover, among the synthesized alloys, Pd_3_M alloy structures were less olefin selective than PdM alloys which were, in turn, almost 100% selective to propylene. This selectivity improvement was interpreted by changes in the DFT-calculated binding energies and activation energies for C–C and C–H bond activation, which are ultimately influenced by perturbation of the most stable adsorption site and changes to the d-band density of states. Furthermore, transition state analysis showed that the C–C bond breaking reactions require 4-fold ensemble sites, which are suggested to be required for non-selective, alkane hydrogenolysis reactions. These sites, which are not present on alloys with PdM structures, could be formed in the Pd_3_M alloy through substitution of one M atom with Pd, and this effect is suggested to be partially responsible for their slightly lower selectivity.

## Abstract 2

Alloying is well-known to improve the dehydrogenation selectivity of pure metals, but there remains considerable debate about the structural and electronic features of alloy surfaces that give rise to this behavior.

## Graphical abstract

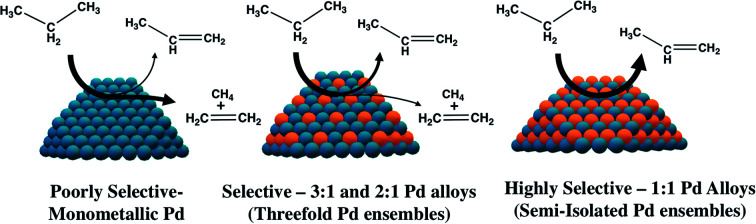

## Introduction

Dehydrogenation is the first step in the activation of alkanes, and on-purpose catalytic dehydrogenation is becoming increasingly important with the widespread exploitation of shale gas reserves, which contain a significant fraction of C_2_+ alkanes.^1^ Further, light olefins such as ethylene and propylene produced from alkane dehydrogenation are important feedstocks for the petrochemical industry.^2,3^ However, dehydrogenation is an endothermic reaction, where high equilibrium conversion is favored by high temperature and low alkane pressure.^4^ At the high temperature required for dehydrogenation of light alkanes, hydrogenolysis also occurs, forming methane and other lower carbon number species leading to low olefin selectivity.^5,6^ Rationally improving dehydrogenation catalyst selectivity, in turn, relies upon development of molecular-level understanding of these unselective hydrogenolysis pathways.

Hydrogenolysis is a structure sensitive reaction, implying that the turnover rate is a function of the surface structure and Pd ensemble size.^7^ Modifications to the ensemble can occur by either changing the particle size or by diluting the active metal with a second inactive metal. In contrast, dehydrogenation is believed to be a relatively structure insensitive reaction, meaning it only requires a single active metal atom to be catalyzed, and hence the dehydrogenation turnover rate shows only modest variations with particle size and ensemble size.^8–10^ To improve the selectivity of dehydrogenation catalysts, it is possible to exploit this difference in structure sensitivity between dehydrogenation and hydrogenolysis through alloying. By separating active metals with an inactive atom, termed a promoter, the turnover rate of hydrogenolysis can be decreased while the turnover rate of dehydrogenation is minimally changed, resulting in increased olefin selectivity.^7^ Most of the early work on structure sensitivity in hydrogenolysis and dehydrogenation reactions was performed on binary alloy systems that form solid solutions.^11^ In these structures, a solute metal randomly substitutes for a solvent metal atom in the parent lattice. This leads to a distribution of active metal ensemble sizes, and high dehydrogenation selectivity is only achieved at very high dilutions of the active metal.^12,13^ Recent work has focused more on intermetallic compounds, which have a fixed (or narrow) composition range and can have crystal structures that differ from their pure components while possessing comparable or higher selectivities than solid solutions.^14^ In these intermetallic compounds, the ensemble size is determined by the crystal structure and atomic plane, and in some materials, such as PtZn, the active metal atoms are completely isolated from one another by promoter atoms.^15^ In other structures, there are still small active metal ensembles, such as in the case of Pt_3_M alloys with the L1_2_ structure.^16,17^ These well-characterized ordered structures of intermetallics are useful in identifying the geometry of active metal ensembles that are required for selective performance of propane dehydrogenation chemistry, and their identification is the primary objective of the present work.

Theoretical studies of dehydrogenation reactions, using density functional theory, have focused mainly on Pt and Pt alloys.^18–26^ Early mechanistic studies on Pt_3_Sn alloys^18,27^ showed that a simple thermodynamic selectivity descriptor (defined as the difference between the propylene desorption and the activation energy barrier of the first deep dehydrogenation reaction) correlates with the observed higher selectivity of Pt–Sn alloys as compared to pure Pt. This descriptor has been used to computationally estimate olefin selectivities for many alloy of Pt.^21,22,26^ In addition, recent work with microkinetic model analysis by Saerens *et al.*^23^ on Pt (111) has shown that, along with the propylene dehydrogenation step, C–C bond breaking of propyne, which is a deeply dehydrogenated derivative of propylene, is also one of the relevant steps for byproduct formation and thereby adversely affects the selectivity of propylene production. Together, these studies suggest that both the simple selectivity descriptor, comparing desorption and further dehydrogenation of propylene, and C–C bond breaking of deeply dehydrogenated species can be important for prediction of selectivity trends across alloys. Further, the transition states of these steps are strongly affected by the geometry and ensemble sizes of active atoms available on the catalyst surfaces.

As discussed above, most research on selective dehydrogenation catalysts has focused on Pt and its alloys, since pure Pt is shown to be more stable against deactivation and to have less hydrogenolysis activity than other metals.^28^ Nonetheless, a few recent reports have also shown that alloys of Pd can have dehydrogenation selectivities comparable to Pt alloys.^29,30^ The ability of promoter metals to suppress hydrogenolysis in alloys in a way seemingly independent of the active metal's intrinsic hydrogenolysis activity motivates systematic study of alloy selectivity not only in Pt alloys, but also in other less selective metals such as Pd. This strategy should give insight into the salient features of alloying that lead to improved selectivity, independent of the behavior of the active metal, and ultimately open up a larger space of catalyst compositions and structures for use as dehydrogenation catalysts. Hence, in this combined experimental and computational study, we synthesize a series of Pd alloy catalysts with five different promoters: In, Zn, Ga, Fe, and Mn. Alloy catalysts were synthesized as to be pure phase and of similar particle size to permit comparisons of selectivity based on differences in promoter identity and the crystal structure of the alloy phase. The formation of alloy phases was verified using *in situ* synchrotron XRD and *in situ* XAS. Catalytic tests show that propylene selectivities are highest for site-isolated alloys (1 : 1), while olefin selectivity of alloys with threefold Pd ensembles is slightly lower, and all of the alloys had significantly higher selectivities than pure Pd. The experimental trends in dehydrogenation selectivity are further compared with calculated trends in adsorbate binding energies and transition state energies for C–C and C–H bond activation. Fully consistent with the experimental results, the DFT calculated trends predict that site isolated alloys have higher olefin selectivity than do alloys without site isolation. Further, the improved olefin selectivity for the 1 : 1 alloys is found to be linked to weaker binding of propylene and to an increase in hydrogenolysis (C–C bond breaking) bond breaking barriers which is driven by the structural dissimilarity of the active ensemble for hydrogenolysis compared to that in pure Pd. In contrast, the active hydrogenolysis ensembles in Pd_3_M alloys are structurally similar to those of Pd, only requiring one atomic substitution to create an ensemble equivalent to Pd. This structural similarity is proposed to be partially responsible for their slightly lower selectivity compared to site isolated alloys.

## Methods

### Catalyst synthesis

Monometallic and bimetallic catalysts were synthesized by sequential loading of the promoter and Pd on Davisil 646 silica (Sigma Aldrich). The loading was done either using incipient wetness impregnation or strong electrostatic adsorption with a calcination step between loadings. All catalysts were reduced after synthesis using a slow ramp through 250 °C and subsequently aged at 550 °C for 30 minutes to reduce the effect of sintering on rate data. The nominal metal loadings for each catalyst are summarized below in Table 1, and full synthetic details are given in the ESI.†

**Table 1:** Synthetic details of Pd and Pd alloy catalysts

Catalyst	1Pd	2Pd	Pd–In	Pd–Fe	Pd–Ga	Pd–Zn	Pd–Mn
Promoter loading (% wt)	—	—	3%	3%	2.5%	3%	5%
Pd loading (% wt)	1%	2%	2%	2%	2.5%	2%	1%

### STEM/EDS

Scanning transmission electron microscopy (STEM) and energy dispersive X-ray spectroscopy (EDS) were performed on an FEI Talos F200X S/TEM with a super-X EDS system and a high brightness field emission electron source. The microscope was operated at 300 keV and STEM images were recorded using a high angle annular dark field (HAADF) detector. Ground catalyst powder was physically mixed with a copper 300 mesh lacey carbon coated TEM grid (SPI supplies). Reported particle size distributions are number average particle sizes and were determined by measuring more than 250 particles. Measurement of particles was done using the FIJI distribution of ImageJ.^31^

### *In situ* synchrotron X-ray diffraction (XRD)

*In situ* synchrotron XRD was performed at the ID-11C beamline of the advanced photon source. XRD was performed in transmission geometry using an X-ray wavelength of 0.1173 Å (105.7 keV). Samples for XRD were ground into a fine powder and pressed into a self-supporting wafer. The sample wafers were then loaded into a water cooled linkam stage capable of gas flow and heating. The X-ray transparent windows on the cell were made of kapton film. The cell was purged with He before a flow of 3.5% H_2_ in He was started and the temperature ramped to 550 °C at 10 °C min^−1^. After a 30 minute dwell at temperature, the cell was cooled to 35 °C and a pattern was collected. Diffracted X-rays were measured using a PerkinElmer large area detector. Detector calibration was performed using a CeO_2_ standard. Trace oxygen was removed from He using an oxygen trap made by Restek.

Detector calibration and pattern integration were performed using GSAS II software.^32^ Background subtraction was done using patterns of the empty cell and the bare SiO_2_ support. Pattern simulation was done using Materials Analysis Using Diffraction (MAUD) and phase references from ICSD.^33–38^ Particle size broadening in simulated patterns was varied by changing the isotropic crystallite size.

### *In situ* X-ray adsorption spectroscopy (XAS)

Pd K edge XAS was performed at the MRCAT 10BM line of the advanced photon source. Samples were measured in transmission mode using three ion chambers which allowed for simultaneous measurement of a Pd foil reference. Samples were ground into a fine powder and pressed inside of a stainless-steel sample holder which was loaded into a quartz tube reactor. The reactor was sealed at each end by a 1 inch ultra-torr union modified with a gas flow port and a kapton window. The reactor was purged with He and then treated at 550 °C in 3.5% H_2_ for 30 minutes. The cell was then purged at high temperature with He to desorb chemisorbed hydrogen and decompose any PdH*_x_* that may have formed during treatment. The samples were then cooled to room temperature in He and scanned. Samples were also scanned after exposure of the reduced samples to air at room temperature.

Extended X-ray adsorption fine structure (EXAFS) data on Pd and Pd alloys were fit using WinXAS software. The extracted chi was *k*^2^ weighted and Fourier transformed over a *k* range of 2.9–12 Å^−1^. Phase and amplitude functions for Pd–Pd scattering were extracted from Pd foil with a coordination number of 12 and a bond distance of 2.75 angstroms. Pd–O scattering was extracted from Pd(OAc)_2_ (4Pd–O bonds at 2.05 Å). Bimetallic scattering (Pd–Mn at 2.62 Å, Pd–Zn at 2.71 Å, and Pd–Ga at 2.6 Å) were constructed using FEFF with the simulated amplitude reduction factor, Debye–Waller factor and *E*_0_ correction fixed to the values fit to Pd foil. Fitting was performed in *R* space on isolated first shell scattering for each sample by allowing the coordination number, bond distance, Debye–Waller factor and *E*_0_ correction to vary.

Reduction–oxidation difference EXAFS was performed by subtracting unweighted chi data of the reduced and room temperature air exposed samples as in ref. 39. The core of the nanoparticle, which is unchanged during the surface oxidation process is subtracted out in the difference, and only the signal from surface scattering modified during the reduction–oxidation process remains. Metal–metal scattering, which are lost during oxidation remains in phase with experimental phase and amplitude functions, while the new Pd–O scattering peak is phase shifted by π-radians with respect to the experimental reference due to the subtraction in the difference spectra. Fitting of the difference spectra was performed in *R* space on Fourier transformed *k*^2^ weighted difference chi. Fourier transforms were taken over a *k* range of 2.9–10 Å^−1^ and fit over an *R* range of 1–3 Å. The fitting procedure was then identical to the above reduced samples.

### Propane dehydrogenation

Propane dehydrogenation was performed in a fixed bed microreactor. 50–150 mg of catalyst was mixed to a total mass of 1 g with davisil 646 silica and loaded into a quartz tube reactor with an inner diameter of 9.5 mm. The catalyst bed was dried in flowing nitrogen at 100 °C for 15 minutes and then reduced in 5% H_2_ in N_2_ at 550 °C for 30 minutes. Before starting the flow of reactant gasses, hydrogen was purged from the bed by flowing 100 cm^3^ of nitrogen for 5 minutes. Propane dehydrogenation reactions were carried out at 550 °C at 3 PSIG with 2.5% propane and 2.5% hydrogen. Conversion was adjusted by changing the total flow rate and mass of catalyst used in a test. Each conversion/selectivity data point was collected on a fresh sample of catalyst from the same synthetic batch. The reproducibility of the conversion for a given catalyst mass was about 3%, with the largest source of error coming from the catalyst mass, which was accurate to within 1 mg. Conversion and selectivity were calculated on a carbon basis of gas phase products measured by an online HP 6890 gas chromatograph with a flame ionization detector and a restek Alumina BOND/Na_2_SO_4_ column. A chromatogram was collected every 5 minutes for 90 minutes. The error in the conversion and selectivity, as estimated from standard deviation of the inlet propane peak area (as determined by flowing through a bypass line) was smaller than the plotted data points. The resulting time on stream data was fit with a first order exponential function to give the selectivity and conversion at zero deactivation.

Propylene production turnover rates were measured at differential conversion (<5%) in 2.5% propane and 2.5% hydrogen at 550 °C. Rates were normalized based on the fraction of exposed Pd determined by surface oxidation EXAFS using the Pd–O coordination number. 100% PdO has a Pd–O coordination number (CN) of 4; thus the dispersion was determined from the fit Pd–O CN divided by 4.

### Density functional theory

Periodic density functional theory (DFT) calculations were performed using the Vienna *Ab initio* Simulation Package (VASP).^40–43^ The Kohn–Sham equations were solved self-consistently using the Perdew, Burke, and Ernzerhof (PBE) functional^44^ with the projected augmented wave (PAW) method.^45,46^ The converged bulk lattice constants for fcc Pd, Pd_3_Fe, and bcc PdIn were *a* = 3.94 Å, *a* = 3.89 Å, and *a* = 3.3 Å, respectively. The lengths of bulk tetragonal PdZn unit cell vectors were *a* = 2.9 Å and *c* = 3.42 Å, while the vectors for the orthorhombic Pd_2_Ga unit cell were *a* = 4.1 Å, *b* = 5.58 Å, and *c* = 7.82 Å. To simulate the fcc (111) surfaces of Pd and Pd_3_Fe, a 3 × 3 × 5 unit cell was used, while to simulate the core–shell structure of a Pd_3_Mn/Pd alloy surface, a 4 × 4 × 5 unit cell was used. For the 1 : 1 alloy surfaces of PdZn (101) and PdIn (110), a 2 × 3 × 5 unit cell was chosen. Finally, for the orthorhombic Pd_2_Ga (010) surface, a 2 × 2 × 6 unit cell was simulated. The bottom two layers have been fixed for all alloy surfaces considered. A planewave energy cutoff of 400 eV was used with a 3 × 3 × 1 Monkhorst–Pack *k*-point grid for Pd (111), Pd_3_Fe (111), PdIn (110), and PdZn (101) alloy surfaces, while a 2 × 2 × 1 *k*-point grid was used for Pd_3_Mn/Pd (111) because of its larger unit cell size, and a 3 × 2 × 1 *k*-point grid was used for Pd_2_Ga (010) due to its orthorhombic unit cell symmetry. These values were confirmed to converge the adsorption energies to within 0.05 eV. The adsorption properties and thermodynamic energy barriers on the surface were calculated using DFT geometry optimizations until the forces were converged within 0.02 eV Å^−2^. To test the effect of van der Waals interactions on the adsorbates, selected binding energy calculations on the Pd (111) and PdIn (110) surfaces were also performed with BEEF-vdW^47^ and optPBE^48^ functionals. All calculations were spin polarized, and dipole corrections were used to cancel out the net dipole moment on the slab. The Methfessel–Paxton scheme was used with an energy smearing of 0.2 eV to determine the partial electron occupancies. Further, a vacuum of 20 Å was used to separate the two slabs in the *z*-direction. The activation barriers were determined with Climbing Image Nudged Elastic Band (CINEB) calculations using both the first and second order methods (QuickMin, LBFGS, and Dimer) developed by Henkelman and coworkers.^49–52^ For the NEB calculations, depending on the length of reaction coordinate, 6–8 images were used, and the images between initial and final states were generated using the Image Dependent Pair Potential (IDPP) method.^53^ Finally, for the Density of States (DoS) calculations, an energy cutoff of 520 eV and a gamma-centered 9 × 9 × 1 *k*-point grid were used in conjunction with the tetrahedron method using Blöchl corrections.

The adsorption energies of open-shell species were calculated by referencing them to the corresponding gas phase closed-shell species energies and adding a stoichiometric amount of gas phase H_2_. Therefore, the binding energies of dehydrogenated C_3_, C_2_, and C_1_ species were estimated using gas phase propane, ethane, and methane energies, respectively. For each of these adsorbates, all the distinct binding configurations were generated using CatKit, a python based open-source framework developed by Boes *et al.*,^54^ and implemented with some modifications for binding of C_3_ adsorbates. The geometry optimizations were then performed for all the sites and configurations, and the unique configurations were identified and databased for each adsorbate and catalyst surface using an in-house algorithm. The energies of the most stable structures were used for the analysis presented in this work.

## Results

### Structural characterization

Detailed characterization of the particle size and phase composition of the Pd–In and Pd–Fe catalysts is detailed in ref. 29 and 30, respectively. Herein, detailed characterization is given only for catalysts newly synthesized for this work: Pd–Ga, Pd–Mn, and Pd–Zn, though PdZn, which has been reported previously, was reproduced according to the synthesis given in ref. 55.

Table 2 shows the number average particle size for the two monometallic Pd catalysts and five bimetallic Pd catalysts. Representative STEM and EDX maps are shown in Fig. S1 of the ESI† and show the high dispersion of metal nanoparticles and the promoter. The 1% Pd catalyst was smaller in size than the 2% Pd catalyst due to the high calcination temperature employed in the synthesis of the latter. The standard deviation of the 2% Pd catalyst was also larger due to the presence of a significant number of large agglomerates, likely resulting from the coalescence and sintering of smaller particles. The bimetallic samples all had particle sizes within one standard deviation of one another and were approximately 1.5–2 nm. Similar to the 2% Pd catalyst, the 2.5%Pd–2.5%Ga catalyst contained both small 1.5 nm particles and larger (5+ nm) agglomerates, which is reflected in the larger standard deviation of the particle size.

**Table 2:** Number average particle size for Pd and bimetallic catalysts

Sample	Number average particle size (nm)
1Pd	1.4 ± 0.5
2Pd	3.7 ± 2.0
2Pd–3Zn	1.5 ± 0.7
2Pd–3In	1.8 ± 0.4
2.5Pd–2.5Ga	2.1 ± 1.6
2Pd–3Fe	1.5 ± 0.7
1Pd–5Mn	1.5 ± 0.5

**Fig. 1:**
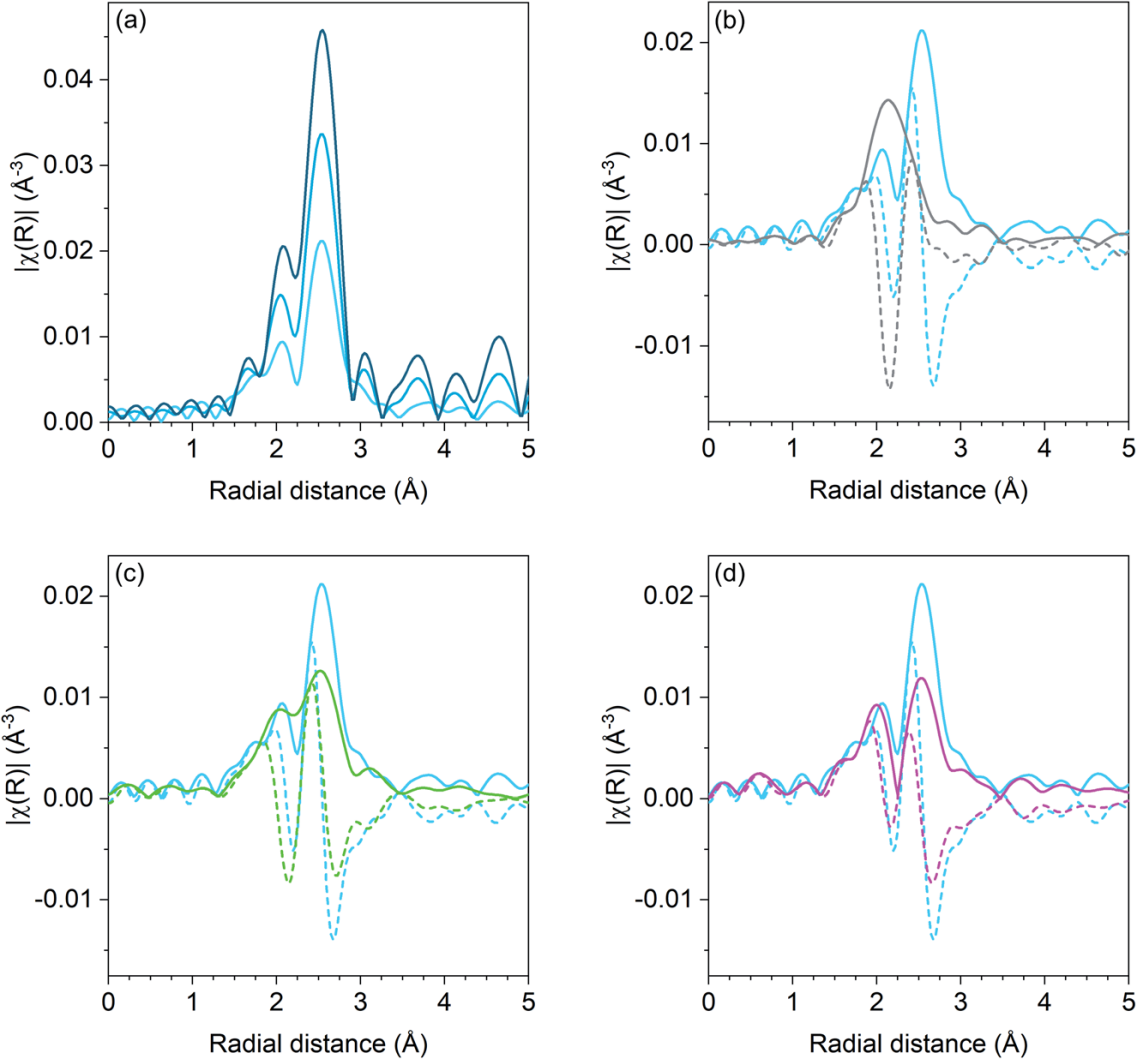
*R* space Pd K edge EXAFS magnitude (solid lines) and imaginary (dashed) components of Pd catalysts. (a) Monometallic Pd catalysts: Pd foil (dark blue), 2Pd (blue), and 1Pd (light blue), (b) 2Pd–3Zn (grey) and 1Pd (light blue), (c) 2.5Pd–2.5Ga (green) and 1Pd (light blue), and (d) 1Pd–5Mn (magenta) and 1Pd (light blue). Spectra were collected at room temperature in He after reduction at 550 °C in 3.5% H_2_.

Pd K edge EXAFS results for the Pd, Pd–Ga, Pd–Mn, and Pd–Zn catalysts are shown in Fig. 1. For the monometallic Pd catalysts, the amplitude of the first shell scattering is attenuated relative to the Pd foil due to the large fraction of surface atoms in the sample which decreases the average coordination number below 12 for bulk fcc metals. No scattering from Pd–O is present, demonstrating that the catalyst is in the metallic state. Fitting the first shell scattering for 1Pd (shown in Table 3) gave a coordination number of 7.7 with a bond distance of 2.74 Å, which is consistent with a Pd nanoparticle. The 2Pd sample had a total coordination number of 10.4 at a bond distance of 2.74 Å.

**Table 3:** EXAFS fitting parameters for Pd foil, Pd, Pd–Mn, Pd–Ga, and Pd–Zn

Sample	Scattering pair	CN	*R* (Å)	Δ*σ*^2^	*E*_0_ (eV)
Pd foil	Pd–Pd	12	2.75	0	0
1Pd	Pd–Pd	7.7	2.74	0.0035	−1.3
2Pd	Pd–Pd	10.4	2.74	0.0020	−1.8
1Pd–5Mn	Pd–Pd	6.1	2.71	0.0050	−2.6
	Pd–Mn	1.4	2.66	0.0050	−1.0
2.5Pd–2.5Ga	Pd–Pd	4.1	2.78	0.0030	−3.6
	Pd–Ga	2.7	2.49	0.0030	−3.7
2Pd–3Zn	Pd–Pd	1	2.81	0.0020	−5.1
	Pd–Zn	4.1	2.54	0.0020	−5.2

The first shell scattering envelope in 2Pd–3Zn, shown in Fig. 1b, changes in shape relative to the monometallic Pd sample due to the formation of Pd–Zn bonds in the catalyst. The single *R* space peak is consistent with Pd having only nearest neighbor Zn. The broadness of the peak occurs due to an overlap between scattering from the first nearest neighbor Zn and single scattering from second nearest neighbor Pd. Fitting (shown in Table 3) gave 4.1 Zn neighbors at 2.54 Å and 1.0 Pd neighbors at 2.81 Å. The short Pd–Zn distance and long Pd–Pd distance is consistent with the structure expected for a 1 : 1 phase.^36^ Two Pd–Zn phases with isolated Pd are known, differing only by a tetragonal distortion which changes the bond distance and coordination number of second nearest neighbor Pd. While the Pd–Pd bond under 3 Å is consistent with the β_1_ phase, the ratio of Pd–Pd : Pd–Zn bonds is lower than expected for this phase (1 : 2), which would be consistent with a mixture of the structurally similar β (cubic CsCl) and β_1_ (tetragonal CuTi) phases, though some departure from the bulk ratio should be expected due to the small particle size.

The first shell scattering envelope from 2.5Pd–2.5Ga bears resemblance to the monometallic Pd catalyst due to the presence of both Pd–Pd and Pd–Ga scattering. Pd–Pd scattering is comprised of three peaks, with the lowest intensity peak at the lowest *R* value and the highest peak at the highest *R* value. In the Pd–Ga catalyst, the second peak is much closer in intensity, relative to the third high *R* peak, as compared to the monometallic Pd sample due to scattering from Pd–Ga. Fitting of the 2.5Pd–2.5Ga catalyst gave a Pd–Pd coordination number of 4.1 at a bond distance of 2.78 Å and a Pd–Ga coordination number of 2.7 at a bond distance of 2.49 Å. The Pd–Ga : Pd–Pd coordination number ratio (0.66) is consistent with the local Pd environment in Pd_2_Ga which has a Pd–Ga : Pd–Pd ratio of 5 : 8 (0.63).

Similar to the Pd–Ga catalyst, the 1Pd–5Mn catalyst also exhibited a change in shape of the first shell scattering envelope, showing two peaks close to the same intensity instead of three peaks of increasing intensity. The scattering resembles that of Pd–Fe,^30^ albeit with the first peak at low *R* being lower in intensity than the high *R* peak. Fitting the Pd–Mn catalyst gave a Pd–Pd coordination number of 6.1 with a bond distance of 2.71 Å and a Pd–Mn coordination number of 1.4 at a bond distance of 2.66 Å, indicating Pd-rich bimetallic particles.

**Fig. 2:**
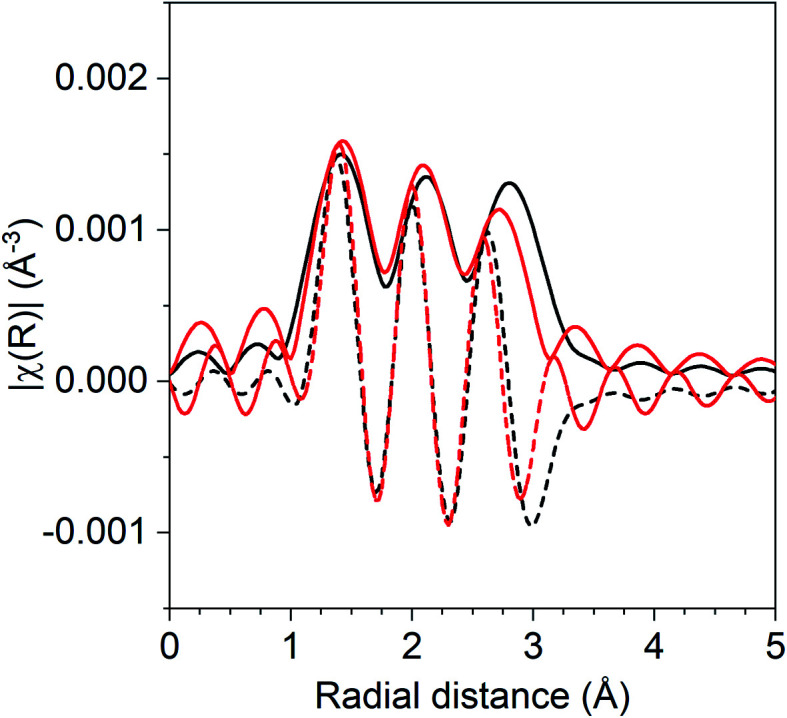
Reduction–oxidation difference EXAFS of 1Pd–5Mn with the experimental difference magnitude (solid) and imaginary (dashed) shown in black and the difference fit shown in red.

The coordination number ratio in 1Pd–5Mn (0.23) does not match that of any bulk Pd–Mn phase. To further understand the structure of the catalyst, reduction–oxidation difference EXAFS was performed to examine the local Pd coordination in the surface layer of the nanoparticle.^39^Fig. 2 shows the *R* space difference XAS spectra between the 1Pd–5Mn catalyst in the reduced state and after exposure to air at room temperature. Three peaks are present: the lowest *R* space peak is due to Pd–O scattering formed during the surface oxidation, while the two higher *R* peaks between 2–3 Å (phase uncorrected distance) are due to the loss of Pd–Mn and Pd–Pd bonds. Table 4 shows the fit of the difference spectra. The Pd–O coordination number was 0.4 at a bond distance of 2.05 Å, a bond distance typical for PdO. The surface Pd–Mn to Pd–Pd ratio from the difference is 0.56, significantly higher than the total nanoparticle ratio. The difference in Pd–Mn to Pd–Pd ratio between the surface and fully reduced catalysts is consistent with a core–shell nanoparticle having a surface phase that is Mn rich with respect to the average particle composition.^39^

**Table 4:** Reduction–oxidation difference EXAFS fit of 1Pd–5Mn

Scattering path	Coordination number	Bond distance (Å)	Δ*σ*^2^ (Å^2^)	*E*_0_ (eV)
Pd–O	0.4	2.05	0.002	2.3
Pd–Pd	0.9	2.73	0.004	−3.2
Pd–Mn	0.5	2.63	0.004	1.8

To determine the phase composition of the catalysts suggested by the EXAFS fits, *in situ* synchrotron XRD was used. A high X-ray energy was used to maximize the difference in structure factor between the amorphous SiO_2_ support and Pd. The high flux provided by an insertion device allows for collection of data with sufficient signal-to-noise to resolve the small broad features from nanoparticles. Use of an *in situ* cell ensures that the entire nanoparticle remains in the metallic state during measurement; otherwise, the surface of the nanoparticle is oxidized during measurement, leading to misleading values of the particle size, NP structure, and lattice parameter.^56^

**Fig. 3:**
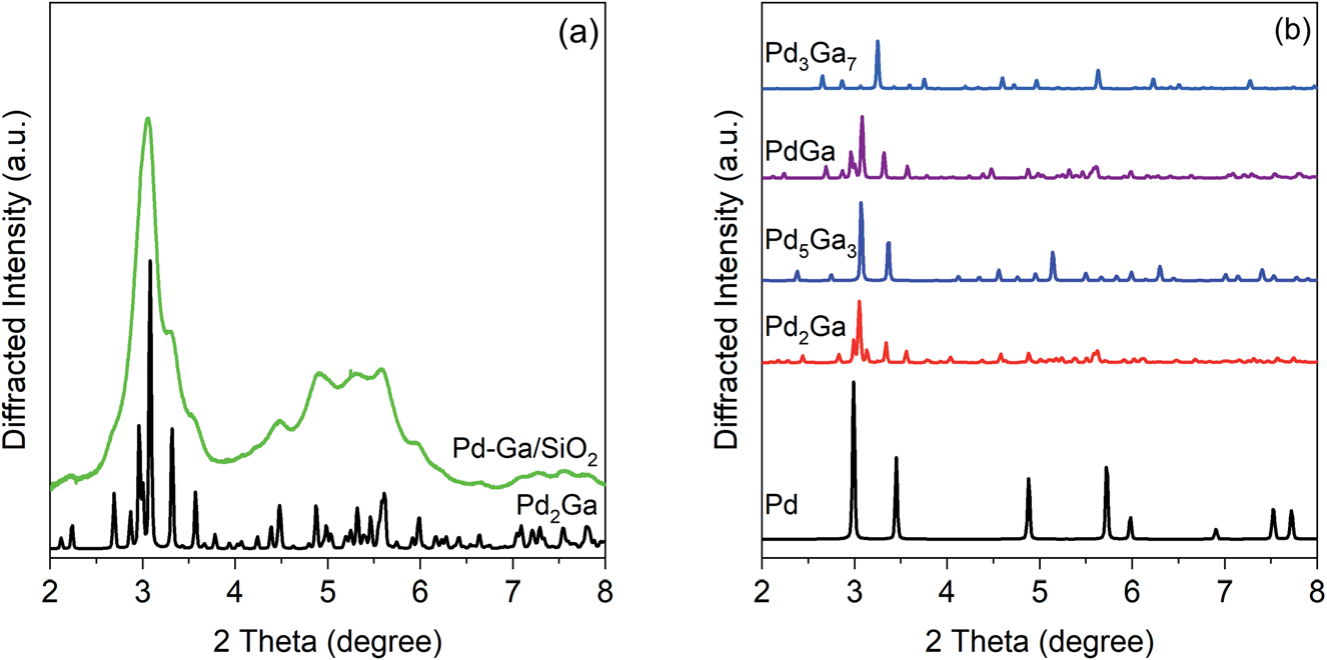
(a) *In situ* synchrotron XRD pattern for 2.5Pd–2.5Ga (green) after a reduction treatment at 550 °C in 3.5% H_2_ with simulated Pd_2_Ga pattern (black), and (b) simulated bulk patterns for Pd–Ga intermetallic compounds.

Fig. 3 shows the simulated patterns of known bulk Pd–Ga phases and the experimental pattern for the Pd–Ga catalyst. The experimental pattern, though significantly broadened due to the small particle size, matches that of Pd_2_Ga, which has an orthorhombic Co_2_Si structure. The pattern is inconsistent with monometallic Pd and other Pd–Ga alloy phases including Pd_5_Ga_3_, PdGa, and Pd_3_Ga_7_. The high X-ray energy used (105.7 keV) causes diffraction peaks to occur at lower 2 theta and over a smaller 2 theta range than is typical of a laboratory XRD instrument. The XRD result is consistent with the EXAFS results which show Pd having a neighbor ratio (Pd–Ga : Pd–Pd) consistent with that of Pd_2_Ga, in which Pd has 5 Ga neighbors and 8 Pd neighbors. Simulated Pd_2_Ga patters with different crystallite sizes (Fig. S2†) estimate a particle size close to 3 nm.

**Fig. 4:**
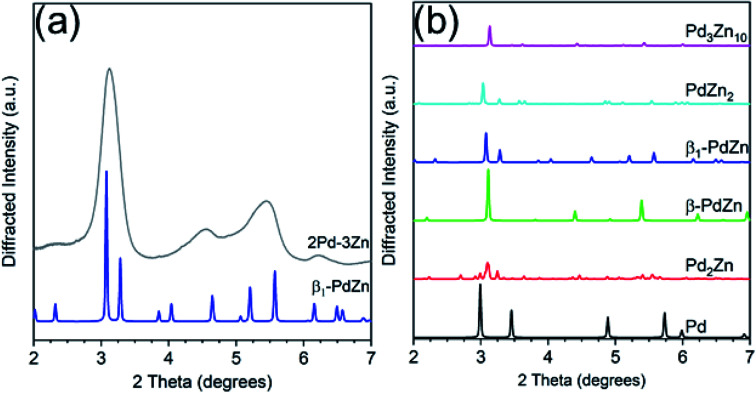
(a) Experimental *in situ* synchrotron XRD pattern for the Pd–Zn catalyst (grey) along with simulated pattern for the β_1_-PdZn phase. (b) Simulated XRD patterns of bulk PdZn phases.

Fig. 4 shows simulations of the bulk Pd–Zn phases and the experimental pattern for 2Pd–3Zn. Similar to the Pd–Ga catalyst, the diffraction peaks in the Pd–Zn catalyst are broadened due to the small particle size. Four distinct peaks are resolved, though some are asymmetric due to peak overlap. The pattern largely matches that of the β_1_-PdZn phase, which has a tetragonal unit cell and body centered symmetry. However, there is some peak misalignment between 4–6 degrees which suggests some distortion from the *a*/*c* lattice parameter ratio expected for bulk β_1_ Pd–Zn (1.147). Evidence of this is seen in the EXAFS bond distances, where the Pd–Pd bond distance is contracted 3.1% from the bulk value, whereas Pd–Zn is contracted 3.8%, giving a *c*/*a* ratio of 1.126. The tetragonal distortion also decreases when Zn partially substitutes at Pd sites, which leads to a cubic CsCl structure (*c*/*a* = 1) and a Pd–Zn/Pd–Pd coordination number ratio higher than 2.^36^ The poor separation and peak asymmetry in the diffraction peaks at 4.55 and 5.45 degrees is more consistent with the β_1_, which has multiple peaks in this region, in contrast to the β phase, which only has one weak peak. However, the β phase may be present as a minor impurity, or the particle composition may be off-stoichiometric (Zn rich) leading to a smaller tetragonal distortion than expected. Monometallic Pd and other non 1 : 1 Pd–Zn phases do not match the measured diffraction pattern of 2Pd–3Zn. The assignment of a 1 : 1 PdZn phase is consistent with the EXAFS results which show only Zn nearest neighbors and a second nearest neighbor Pd at a long bond distance. Previous reports of Pd–Zn bimetallics synthesized by sequential incipient wetness impregnation have also shown the formation of the β_1_-PdZn phase.^57^

### Propane dehydrogenation

**Fig. 5:**
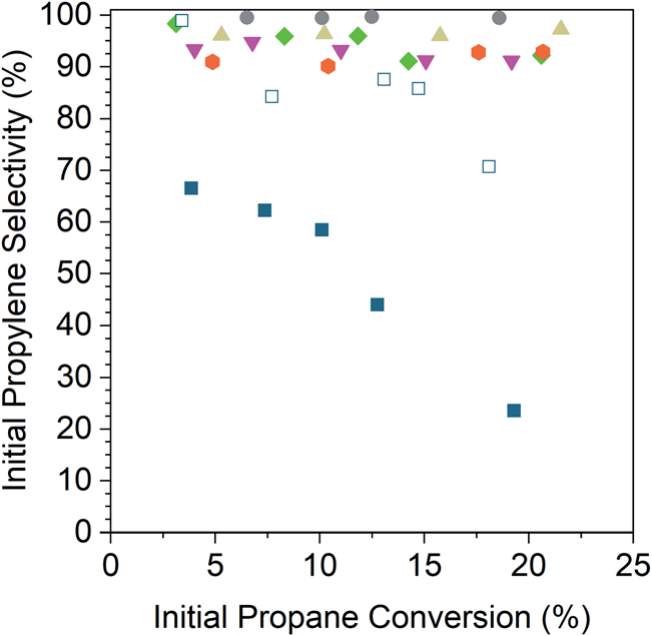
Initial propylene conversion for 2Pd–3Zn (grey circles), 2Pd–3In (tan triangles), 2.5Pd–2.5Ga (green diamonds), 1Pd–5Mn (magenta inverted triangles), 2Pd–3Fe (orange hexagons), 1Pd (open dark blue squares), and 2Pd (filled dark blue squares) plotted against the initial propane conversion. Tests were performed at 550 °C in 2.5% H_2_ and 2.5% propane after pre-reducing at 550 °C in 5% H_2_.

Fig. 5 shows the initial propylene selectivity at different levels of initial propane conversion for Pd and Pd alloy catalysts reported on a carbon basis of gas phase products. Catalyst tests were performed with cofed hydrogen as a more demanding test of catalyst selectivity, since the latter is required for hydrogenolysis. It was also necessary to cofeed hydrogen to improve the stability of the monometallic Pd catalysts which otherwise deactivated too rapidly to properly extrapolate conversion and selectivity to zero time on stream. A 1 : 1 ratio of propane to hydrogen gave deactivation rate constants (Table 5) of the same order of magnitude for Pd and Pd alloy catalysts which allowed for initial conversion and selectivity to be properly determined. The main products were propylene, methane, ethane and ethylene, the latter three resulting from hydrogenolysis. For all alloy catalysts, the carbon balance was in excess of 98% for all tests, and hence the contribution of coke formation to selectivity has been neglected. For the Pd catalysts, the carbon balance decreased from 98% at <5% conversion to 83% at 20% conversion. For consistency, the selectivity and conversion for both Pd catalysts is reported for gas phase products, though this overestimates the selectivity at high conversions. The size of the plotted data points in Fig. 5 is larger than the error bars for conversion and selectivity. Plots of the conversion *vs.* catalyst mass in the differential regime were linear and gave an intercept value of 0% conversion to within 3%.

**Table 5:** Dehydrogenation rate, first order deactivation rate constants, and activity loss after 90 minutes of reaction for Pd and Pd alloy catalysts

Sample	Dispersion	Propylene TOR (s^−1^)	Selectivity at 20% conversion	Deactivation rate constant (min^−1^)/10^−3^	Activity loss after 90 minutes (%)
2Pd–3Zn	0.13	0.30	100	3.3	52
2Pd–3In	0.15	0.25	96	2.3	39
2.5Pd–2.5Ga	0.08	0.20	92	4.0	64
2Pd–3Fe	0.08	0.40	91	6.0	62
1Pd–5Mn	0.10	0.26	91	6.3	48
2Pd	0.18	0.03	25	3.2	54
1Pd	0.35	0.05	70	8.6	80

The 2% Pd catalyst (4 nm) shows poor selectivity, 70%, at differential conversion, and as the conversion is increased, the selectivity quickly drops to around 25% at 20% conversion. The 1% Pd catalyst (1.5 nm) shows much higher selectivity at equivalent conversion compared to the 2% Pd catalyst which demonstrates the effect of particle size on the rate of hydrogenolysis. The selectivity of the 1% Pd catalyst is high (∼100%) at differential conversion but decreases with increasing conversion, falling to 70% at 20% conversion.

In comparison, the alloy catalysts have selectivity above 90% across the conversion range tested, and there is little change in selectivity with increasing conversion. For 2Pd–3Zn, the selectivity was above 99% up to 20% conversion. The 2Pd–3In catalyst selectivity also did not decrease with conversion, but the selectivity was slightly lower than the 2Pd–3Zn catalyst at 96%. The 2.5Pd–2.5Ga catalyst showed a small decrease in selectivity, falling from 98% at 3% conversion to 92% at 20% conversion. The 1Pd–5Mn catalyst had a lower selectivity than the Pd–Zn and Pd–In catalysts, between 95–91%. The 2Pd–3Fe catalyst also did not decrease in selectivity with increasing conversion, but the selectivity was constant at a lower value (91%).

In order to measure dehydrogenation turnover rates, the percentage of surface Pd was measured by surface oxidation difference XAS. Exposing the fully reduced catalyst to air at room temperature causes the surface Pd to oxidize, which can be seen by XAS as Pd–O scattering. In catalysts containing both metallic and Pd–O scattering, the Pd–O coordination number represents the phase fraction of surface Pd which is accessible to oxygen weighted by the natural coordination number of PdO, which has 4 Pd–O bonds. Thus, the Pd–O coordination number divided by 4 gives the palladium dispersion without the confounding effects which would occur in H_2_–O_2_ titration or CO chemisorption caused by absorption or redox of the promoter. Fits of the oxidized catalysts are shown in Table S1 of the ESI.† The method closely matches the dispersion measured by CO chemisorption for the Pd–In catalyst as reported previously.^29^

Table 5 shows the propylene turnover rate for the Pd and Pd alloy catalysts. The monometallic Pd catalysts have a low turnover rate: 0.03 s^−1^ for the 2Pd catalyst, which has a large particle size, and 0.05 s^−1^ for the 1Pd catalyst, which has the same particle size as the alloy samples. The alloy catalysts have propylene turnover rates 4–8 times higher than the monometallic Pd catalyst of the same size. Between the alloys, the propylene turnover rates only varied by a factor of two.

### Density functional theory

To further understand the catalytic trends in the different alloys considered, density functional theory (DFT) calculations were carried out on model surfaces of each alloy. These calculations were performed on the most stable terrace surfaces corresponding to the respective bulk alloy structures found by XAS and XRD experiments. While XAS and XRD are bulk techniques, the average metal particle size in all catalysts was around 2 nm, and as such, a significant fraction of the scattering originates from surface atoms. Further, if any surface segregation occurred, it would be reflected in the Pd–Pd : Pd–M coordination number ratio, which would increase to a value above that of the intrinsic ratio of the respective alloy phase. In other noble metal-containing intermetallic compound catalysts, it has been demonstrated by surface oxidation difference XAS that the particle surface is composed of the alloy phase, even when core shell structures are present.^39,58^ Therefore, for alloy phases with fcc structures (Pd, Pd_3_Fe), the (111) facet was used, for the body centered cubic (bcc) PdIn, the (110) surface was used, and for body centered tetragonal β_1_-PdZn, the (101) surface was used. In all cases, the surface composition was assumed to be equal to the bulk composition. Pd_2_Ga has a low symmetry orthorhombic crystal structure composed of two symmetrically equivalent flat layers stacked along the 〈010〉 direction. These (010) terrace surfaces, which have a stoichiometry matching the bulk, were analyzed, and the same surface has been modeled previously for semi-hydrogenation of acetylene.^59^ There are two crystallographically distinct Pd sites on Pd_2_Ga (hereafter denoted Pd_1_ and Pd_2_), whereas all other alloys have a single crystallographic Pd site. To simulate the core shell structure of Pd_3_Mn/Pd, Pd atoms were replaced with Mn atoms in the first two atomic layers of the slab, corresponding to the ordering of Pd_3_Mn with AuCu_3_ structure that is consistent with the surface EXAFS CN ratio. We note, that although the absolute values of turnover rates will vary between the terraces and high-index surfaces, such as steps, the trends in binding energies, activation energies, and selectivities between alloys are likely to be similar on high-index surfaces, assuming that Pd ensemble sizes are similar to the terraces.

**Fig. 6:**
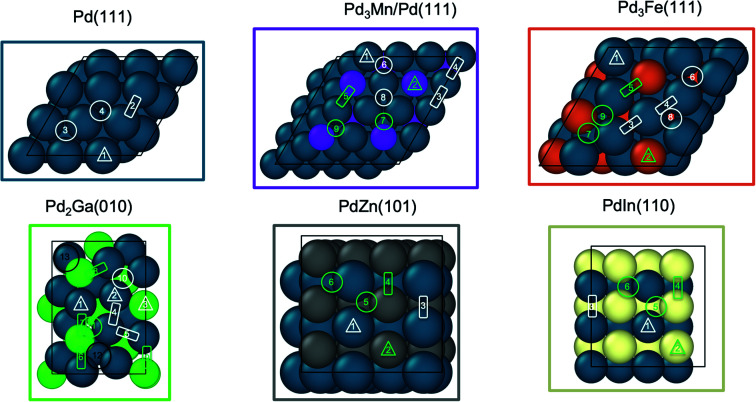
Adsorption sites for Pd and Pd alloy surfaces. Ontop sites are denoted by triangles, bridge sites by rectangles, and adsorption sites involving three or more atoms by circles. Sites marked in white involve direct bonding only to Pd atoms, while those in green indicate that the site involves a promoter atom. The black circles in Pd_2_Ga indicate bonding at a fourfold site.

Fig. 6 shows the binding sites for the slab models. For monometallic Pd, four adsorption sites were considered: onefold ontop, twofold bridge, threefold hcp, and threefold fcc. In the fcc alloys, additional sites including promoter ontop, Pd–M bridge, and threefold sites containing two Pd and one promoter atom were also considered. As the ratio of alloy composition changes from 3 : 1 to 1 : 1, the Pd atoms become completely surrounded by the promoter atoms, where Pd atoms now have promoter atoms as their only nearest neighbors (Zn, In). This leads to loss of Pd-only threefold sites for the 1 : 1 alloy surfaces, while all other sites are the same as the adsorption sites on 3 : 1 fcc alloys. Due to the low symmetry of the Pd_2_Ga (010) surface, a relatively large number of distinct adsorption sites exists. On this surface hollow sites can additionally be formed by four atoms, leading to a total of three ontop sites, five bridge sites, two threefold hollow sites and two fourfold sites.

### Adsorption energies

Adsorption energies and geometries of selected intermediates, including propylene, hydrogen, and ethylidyne, are reported in Tables 6 and 7. These species are representative of both propylene and deeply dehydrogenated intermediates in the PDH reaction network (corresponding results for propyne, propynyl, methylidyne, and carbon are reported in Table S3†). As discussed below, the energetics of these species elucidate trends in propylene selectivity across the space of considered Pd alloys.

**Table 6:** Binding energies of adsorbates on Pd and Pd alloy surfaces, with more positive values indicating weaker adsorption. The binding energies of open-shell species (hydrogen, ethylidyne), are referenced to the corresponding gas phase species (H_2_, ethane)

Adsorbate binding energy (eV)	Pd (111)	Pd_3_Mn/Pd (111)	Pd_3_Fe (111)	Pd_2_Ga (010)	PdZn (101)	PdIn (110)
Propylene	−0.79	−0.73	−0.49	−0.48	−0.13	−0.06
Hydrogen	−0.63	−0.55	−0.46	−0.43	−0.25	0.03
Ethylidyne	0.96	1.35	1.54	1.98	2.96	3.18

**Table 7:** Most stable binding sites of adsorbates on Pd and Pd alloy surfaces. The numbers in parentheses refer to sites as labeled in Fig. 6

Adsorbate binding site	Pd (111)	Pd_3_Mn/Pd (111)	Pd_3_Fe (111)	Pd_2_Ga (010)	PdZn (101)	PdIn (110)
Propylene	Pd–Pd bridge (2)	Pd-bridge (3)	Pd ontop (1)/Pd–Pd bridge (3)	Pd ontop (2)	Pd ontop (1)	Physisorbed
Hydrogen	fcc (4)	Pd–Pd–Pd hcp (6)	Pd–Pd–Pd hcp (6)	Pd–Pd–Pd hcp (10)	Pd–Pd bridge (3)	Pd–Pd bridge (3)
Ethylidyne	hcp (3)	Pd–Pd–Pd hcp (6)	Pd–Pd–Fe fcc (9)	Pd–Pd–Pd hcp (10)	Pd–Pd–Zn hcp (5)	Pd–Pd–In hcp (5)

The adsorption energies of all the intermediates considered are weaker on the Pd alloys as compared to monometallic Pd. Propylene is most stable on the bridge site on the Pd (111) surface (binding energy of −0.79 eV), where it takes a di-sigma configuration (Fig. S6†). Compared to gas phase propylene, the carbon–carbon double bond is elongated from 1.34 Å to 1.45 Å, indicating that the propylene π bond is significantly weakened upon adsorption. On Pd_3_Mn/Pd, the binding energy weakens slightly to −0.73 eV, while the most stable adsorption geometry remains disigma. The binding of propylene is weakened by 0.3 eV on Pd_3_Fe and Pd_2_Ga surfaces, while for the site isolated alloys PdZn and PdIn, the binding is weakened by 0.66 eV and 0.73 eV, respectively. On Pd_3_Fe, propylene binds to both ontop-Pd and bridge Pd–Pd with similar stability, whereas the most stable configuration shifts from bridge Pd–Pd to ontop-Pd for the two post-transition metal alloy surfaces, Ga and Zn. On the PdIn (110) surface, propylene is physisorbed. These results show that the propylene binding energies and adsorption geometries are strongly affected by increases in the promoter content of the alloys.

The most stable adsorption site for the hydrogen atom on pure Pd (111) is the threefold fcc site. The binding energy of hydrogen is weakened by 0.1 eV on core–shell Pd_3_Mn/Pd, while the corresponding weakening is approximately 0.2 eV on Pd_3_Fe and Pd_2_Ga surfaces. The most stable adsorption site for hydrogen is the hollow site composed of three Pd atoms for all alloy surfaces that contain such sites (Pd_3_Mn, Pd_3_Fe and Pd_2_Ga). Similar to the case of propylene, the weakening in H binding for the 1 : 1 PdZn and PdIn surfaces, which do not contain threefold Pd sites, is even larger (0.38 eV and 0.66 eV, respectively), and the most stable binding site shifts to bridge Pd–Pd. Moreover, at the dehydrogenation reaction temperatures (823 K), the adsorbed hydrogen will very likely be in quasi-equilibrium with hydrogen in the gas phase for all of the surfaces considered. Hence, the trends in binding energies will track with the coverages of dissociated hydrogen on the surface, with the lowest coverages found for the 1 : 1 alloy surfaces.

Ethylidyne, a dehydrogenated C_2_ intermediate and product of propyne and propynyl hydrogenolysis, is most stable on the threefold hcp site on the Pd (111) surface (Fig. S7†). The binding energy is weakened by 0.39 eV, 0.58 eV, and 1.02 eV for Pd_3_Mn/Pd, Pd_3_Fe, and Pd_2_Ga terraces, respectively. Ethylidyne is adsorbed on threefold Pd hcp sites on Pd_3_Mn/Pd and Pd_2_Ga, but on the Pd_3_Fe terrace, the site preference changes to the fcc site that contains an Fe atom and two Pd atoms (adsorption at the hcp hollow site composed of three Pd atoms is less favorable by 0.1 eV). The weakening in adsorption energies is considerably larger for the 1 : 1 PdZn and PdIn surfaces, at 2.22 and 2.00 eV, respectively. On these alloys, ethylidyne is most stable on the hcp Pd_2_X sites where the unfavorable interaction of the adsorbate with the promoter atom (In, Zn) contributes to the weakened adsorption. The binding energy trends for propyne, propynyl, methylidyne and carbon on the alloy surfaces (Table S3†) are similar to those observed for ethylidyne.

The unusual binding geometry of ethylidyne, which involves partial coordination to surface Fe atoms on the Pd_3_Fe alloy surface, can be straightforwardly understood in terms of the affinity of deeply dehydrogenated carbon-containing species for Fe. To illustrate this point, adsorption energies on the bcc Fe (110) terrace surface (Table S4†) have also been calculated. The binding of ethylidyne, along with other deeply dehydrogenated intermediates, was found to be 0.5–1 eV stronger than on Pd (111).

The binding energies of the C_3_ species on Pd (111) and PdIn (110) were also calculated using vdW functionals, including BEEF-vdW and optPBE (Tables S6 and S7†). The results show that the addition of vdW corrections across the adsorbates leads to approximately constant shifts in binding energies. Hence, the reaction energetics, and therefore the selectivity trends, obtained using the PBE functional will not significantly change with the consideration of vdW interactions between adsorbates and the surface. The trends in binding energy shifts are also influenced by electronic changes in the Pd atoms, consistent with the d-band model of metal and alloy surfaces,^60,61^ where the Pd alloys with the Pd d-band center shifted furthest from the Fermi level (PdZn, PdIn) showed the largest shifts in adsorption energies. The first and second moments of the d-band for Pd and Pd alloys are given in Table S2.† Experimental evidence for electronic modification of Pd was seen in the Pd L_3_ and K edge XANES of the catalysts, which showed small changes in the edge energy and white line shape. In particular, the L_3_ edge XANES, which probes the 4d and 5s unfilled states, gives direct evidence of this electronic modification which manifests as a change in the whiteline shape in the alloys relative to Pd. The modifications are a result of the heteroatomic bonds in each alloy which modify the Pd density of states, as was demonstrated computationally. Pd L_3_ edge XANES spectra are given in Fig. S3 of the ESI.†

### C–H and C–C bond activation

**Fig. 7:**
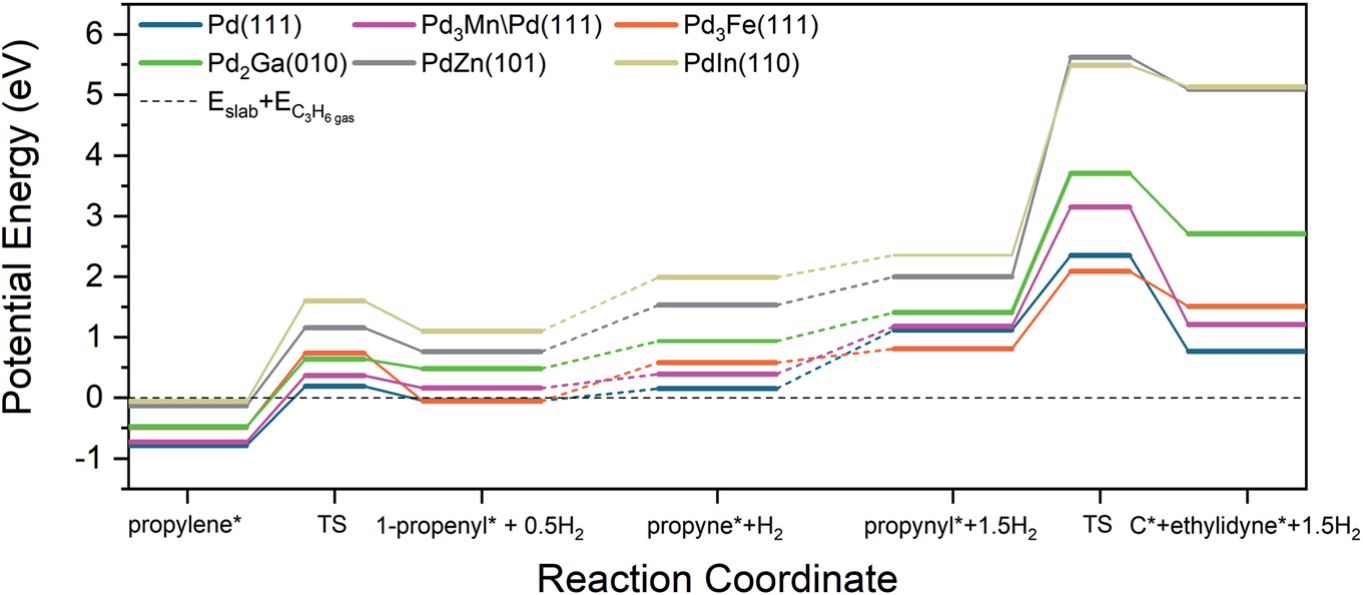
Reaction coordinate diagram for propylene dehydrogenation and propynyl hydrogenolysis.

A commonly used selectivity descriptor for alkane dehydrogenation reactions is the energy difference between the alkene desorption energy and the alkene dehydrogenation barrier, which has been linked to the selectivity trends for propane dehydrogenation on platinum alloys.^27^ Propylene dehydrogenation barriers have been estimated on all the Pd alloy surfaces and are depicted in Fig. 7 and Table 8. The dehydrogenation barrier is seen to increase as the promoter content in the alloy increases. The increase in barrier, as compared to Pd (111), is highest for the PdIn surface (∼0.66 eV increase), while the smallest change is on Pd_3_Mn/Pd (0.1 eV increase). The transition states for these C–H bond breaking steps are shown in Fig. S8.† On all of the alloy surfaces, the dissociated H atom is on the ontop site of Pd, which then shifts to either a hollow site or a bridge site at the final state, depending on the alloy composition. On the other hand, the product propenyl species are close to their most stable sites (a hollow site or a bridge site) and only require a small rotation towards their most stable configurations in the final state. Hence, the reaction coordinate involves a three atom Pd ensemble for the pure Pd, 3 : 1, and 2 : 1 Pd alloy surfaces, while a one atom Pd site is required on the 1 : 1 alloy surface.

**Table 8:** Propylene dehydrogenation activation energy barrier, propylene selectivity descriptor, and activation energy barriers for C–C bond cleavage of propynyl for Pd and Pd alloys

Surface	Propylene dehydrogenation barrier (eV)	Selectivity descriptor (eV)	Propynyl C–C bond breaking barrier (eV)
Pd (111)	0.98	−0.19	1.23
Pd_3_Mn/Pd (111)	1.10	−0.37	1.97
Pd_3_Fe (111)	1.23	−0.74	1.28
Pd_2_Ga (010)	1.12	−0.64	2.30
PdZn (101)	1.29	−1.16	3.62
PdIn (110)	1.66	−1.60	3.13

An approximate selectivity descriptor was calculated from the difference between the propylene adsorption energy (Table 6) and the propylene dehydrogenation barrier (Table 8). More negative selectivity descriptor values indicate more favorable energetics for propylene desorption compared with further dehydrogenation. As the promoter content increases, the selectivity descriptor becomes more negative, suggesting higher selectivity on the indicated alloys. Monometallic Pd had the most positive value of the selectivity descriptor, corresponding to the lowest predicted selectivity, in line with experimental results. In comparison, the Pd_3_Fe and Pd_2_Ga alloys have more negative values (the change is ∼0.5 eV in comparison to Pd) which are reflected in the measured increase in selectivity to 92%. Interestingly, for the core–shell Pd_3_Mn/Pd alloy, the selectivity descriptor increases by only 0.2 eV compared to Pd, but the selectivity increase is comparable to the other 3 : 1 alloy, Pd_3_Fe. Finally, the largest negative values of the selectivity descriptor are for the 1 : 1 PdIn and PdZn alloys (the change is ∼1 eV in comparison to Pd), in agreement with highest selectivities (96, 100%) obtained in our experiments. In general, the primary contributor to these trends in the selectivity descriptor is the change in binding energies of propylene. Changes in C–H bond breaking barriers are smaller; in all alloys except PdIn, the propylene dehydrogenation barriers differ from that of Pd by less than 0.3 eV.

In addition to the selectivity descriptor introduced above, recent work from Saerens *et al.* on Pt (111) suggests that C–C bond breaking in C_3_ species formed from deep dehydrogenation of propylene is also kinetically relevant for unselective formation of byproducts, including methane, ethane, and ethylene.^23^ To probe the significance of these reactions for Pd and Pd alloys, the C–C bond breaking barriers of propyne (C_3_H_4_) and propynyl (C_3_H_3_) on the alloy and pure metal surfaces were calculated. The trends are very similar for both intermediates, and only the analysis for propynyl is discussed in the main text (the corresponding analysis for propyne is presented in Table S5†).

C–C bond activation in propynyl leads to the formation of carbon (C_1_) and ethylidyne (C_2_) species which can further hydrogenate to form methane and ethane, respectively. Alternatively, the adsorbed C_1_ and C_2_ species can dehydrogenate or polymerize to form coke on the surface.^62^ The trends in barriers (Table 7) show that, as the promoter content increases, the C–C bond breaking barrier also increases, except for Pd_3_Fe (111), where the barrier is closer to that on the Pd (111) surface. This smaller change in barrier for Pd_3_Fe can be attributed to the favorable binding of deeply dehydrogenated species in the threefold Pd_2_X sites.

The transition states for propynyl C–C bond breaking for all the considered alloy surfaces (Fig. S9†) show that the product ethylidyne and carbon species are close to each other and are in the process of shifting towards their stable configurations on adjacent hollow sites. For the alloy surfaces, due to the extended nature of transition state, either carbon or ethylidyne must bond with a hetero-promoter atom at the transition state. Indeed, it appears that a four or five surface atom ensemble is involved at the transition state, which is in contrast to the C–H bond breaking in propylene (discussed above), where a one or three atom ensemble was involved. This increase in the number of surface ensemble atoms associated with the transition state, and the consequent contact between the transition state structure and heteroatoms on the alloys, offers an explanation for the larger increase in the C–C bond breaking barriers on alloy surfaces in comparison to the corresponding increases in C–H bond breaking barriers. A reaction coordinate of all selectivity-relevant elementary steps, including C–H and C–C bond cleavage, is shown in Fig. 7.

## Discussion

### Catalyst structure

**Fig. 8:**
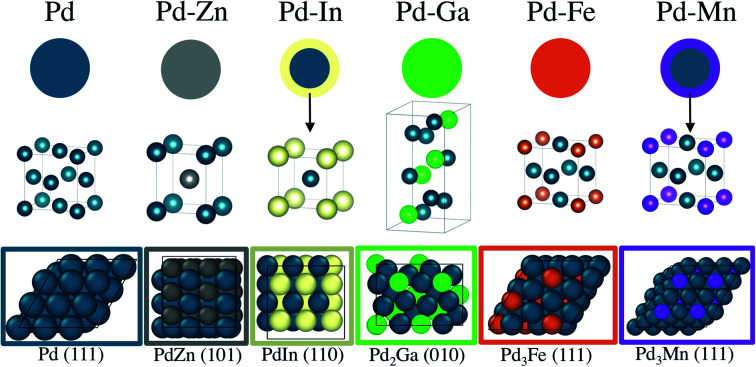
Structural model of Pd and Pd alloy catalysts. The PdIn and PdMn catalysts have alloy shells with a Pd core, while the PdZn, PdGa and PdFe catalysts are pure phases. The unit cell of each alloy structure and the lowest energy surface are pictured beneath the respective nanoparticle models.

The catalyst structures of 2Pd–3Fe and 2Pd–3In are detailed in ref. 29 and 30. The 2Pd–3Fe catalyst is a pure phase Pd_3_Fe alloy, based on the *in situ* synchrotron XRD pattern and matching atomic Pd environment measured by XAS. The Pd–In catalyst had a Pd core and alloy shell of the cubic PdIn phase with a CsCl structure. The XRD pattern for 2Pd–3Zn showed that Pd and Zn form the β_1_-PdZn alloy phase, though it may contain an impurity phase of the closely related β phase or be slightly Zn rich. The formation of the β_1_-PdZn phase in nanoparticle bimetallic Pd–Zn catalysts has been reported and is consistent with the present results.^55,57^ Consistent with the XRD result, the Pd K edge EXAFS showed exclusively Pd–Zn nearest neighbors and Pd–Pd scattering at an elongated distance. The 2.5Pd–2.5Ga catalyst was verified to form the Pd_2_Ga phase with the Co_2_Si structure, consistent with other literature reports on Pd–Ga bimetallics.^63^ The Pd K edge EXAFS reflected the Pd_2_Ga structure in the Pd–Ga : Pd–Pd coordination number ratio which closely matched that of Pd in Pd_2_Ga. The 1Pd–5Mn formed a Pd–Mn bimetallic, as shown by EXAFS, but the low Pd loading and small particle size did not allow for collection of XRD spectra, even by synchrotron XRD. The catalyst was verified to have a core shell structure by difference XAS, which showed that the particle shell is Mn rich with respect to the total particle composition. The Pd–Mn/Pd–Pd neighbor ratio in the nanoparticle matched that of Pd_3_Mn, which has an AuCu_3_ structure. The formation of a shell layer of intermetallic alloy also occurred in 2Pd–3In, and has been reported for other nanoparticle alloy systems.^64,65^ Note that all of the reported alloys are known thermodynamic phases, and as such are expected to be stable structures. The order–disorder transition temperatures for the reported alloys are well above the propane dehydrogenation reaction temperature.^66^ The structural model, unit cell, and slab model of each catalyst is summarized in Fig. 8.

### Selectivity trends in Pd alloys

Testing each catalyst under equivalent propane dehydrogenation conditions clearly distinguished between the rate and selectivity of Pd and the alloys, and showed a smaller difference between the selectivity of alloys with and without Pd site isolation. Although all Pd alloy catalysts are significantly more selective than monometallic Pd, there are differences in selectivity depending on the structure and number of adjacent Pd atoms in the surface ensembles. Among the alloy catalysts, the site isolated alloys β_1_-PdZn and PdIn have the highest dehydrogenation selectivity. The selectivity of the Pd_2_Ga, which has distorted threefold sites where the three atoms are no longer equidistant from one another (as is the case with Pd and the Pd_3_M alloys), was close to that of the 3 : 1 alloys. The Pd_3_Fe and Pd_3_Mn alloys, with threefold Pd ensembles, show the lowest selectivity among the alloys. The difference in selectivity from the most selective alloy (PdZn) and the least selective alloys (Pd_3_Mn, Pd_3_Fe) was approximately 10%, with all alloys showing selectivity at 20% conversion above 90%. The initial dehydrogenation turnover rate for the alloys only varied by a factor of two, and such a small difference should be considered within the error of reproducibly determining turnover rates in a reaction with fast deactivation.^67^ Because all the dehydrogenation turnover rates for the alloys are similar, and carbon balances for the alloys showed a negligible amount of coke formation, the difference in selectivity comes from changes in the rate of hydrogenolysis, which we define as all elementary steps involving reaction of propylene or more deeply dehydrogenated intermediates. An increase in selectivity from 90 to 99% requires that the rate of hydrogenolysis decrease by about an order of magnitude, with the dehydrogenation rate being constant. Thus, while the difference in selectivity between the site isolated alloys and those without is moderate, the difference in the hydrogenolysis rate is large.

As the hydrogenolysis rate largely determines the selectivity ranking of the alloys, DFT modeling efforts were focused on two different steps in the reaction. First, as discussed above, the previously established selectivity descriptor involving the propylene dehydrogenation barrier was determined (Table 8). The dehydrogenation of propylene is the first step in the hydrogenolysis reaction network, and the selectivity descriptor compares the relative favorability of propylene dehydrogenation and propylene desorption. Alloys with the highest promoter content (PdIn, PdZn) had the most negative value of the selectivity descriptor (predicting favorable propylene desorption over dehydrogenation), while the magnitude was lower for alloys with lower promoter content. Although the highest negative value of the selectivity descriptor is for the PdIn alloy, the experimentally observed selectivity is highest for PdZn. Additionally, the 0.2 eV decrease in the selectivity descriptor value from Pd to Pd_3_Mn causes an increase in selectivity from 70% to 91%, yet a further 0.4 eV decrease from Pd_3_Mn to Pd_3_Fe does not alter the selectivity. These results suggest that the selectivity descriptor can qualitatively describe the trends in observed experimental selectivity, but the value should not be used to quantitively rank alloys with similar structures. It can, however, clearly distinguish the selectivity changes observed experimentally between 3 : 1 and 2 : 1 (Pd-rich) alloys (91–93%) and the 1 : 1 (site-isolated) alloys (96–100%).

Another important elementary step in hydrogenolysis is the C–C bond cleavage step. C–C bond cleavage is irreversible and often assumed to be rate-limiting for hydrogenolysis.^6,68,69^ The propynyl C–C bond breaking barriers calculated across the various Pd alloys have similar trends compared to the selectivity descriptor involving propylene dehydrogenation. The largest increase in barriers (corresponding to highest predicted selectivity for propylene production) is found for the 1 : 1 Pd alloys, and the barriers become smaller with decrease in promoter content for non-catalytic promoters. However, in contrast to the trend in the selectivity descriptor on 1 : 1 alloys, PdZn has the largest C–C bond breaking barrier, which is almost 0.49 eV greater than PdIn. This larger barrier on PdZn is also consistent with the greater selectivity observed in the experiments for the PdZn (100%) alloy than the PdIn alloy (96%). In aggregate, the trends in both C–C bond breaking barriers and the selectivity descriptors show good qualitative agreement with experimentally-determined selectivity patterns.

**Fig. 9:**
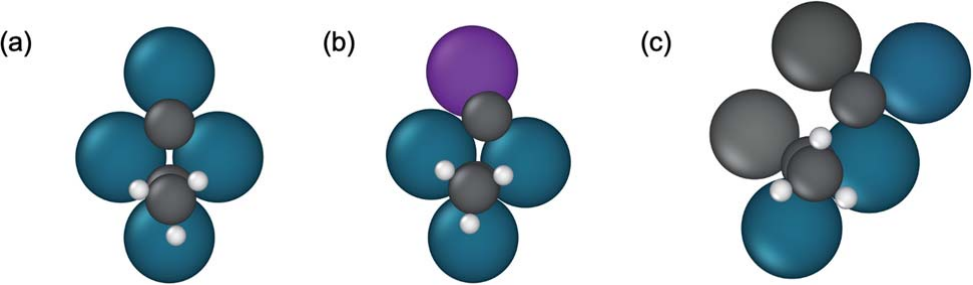
Representative ensembles of surface atoms involved in propynyl C–C bond breaking with corresponding transition state structures. (a) Pd (111) (b) Pd_3_Mn/Pd (111) (c) PdZn (101).

Additional insights into the factors driving propylene selectivity can be obtained by comparing differences in C–C bond breaking transition state structures across the various alloys (Fig. S9†). On pure Pd, the 3 : 1 alloys, and Pd_2_Ga, hydrogenolysis occurs on two adjacent threefold sites across four atoms in a diamond shape (Fig. 9a and b). On the 3 : 1 and 2 : 1 alloy surfaces, this structure implies that the promoter element interacts with one of the dissociated product species (carbon or ethylidyne). In contrast, for the 1 : 1 alloys, the transition state for C–C bond breaking is slightly more extended, involving a five atom ensemble of which two atoms are promoter elements. Hence, in this case, both the dissociated carbon and ethylidyne interact with the promoter atoms (Fig. 9c). This larger interaction of the transition state with the promoter atoms for the 1 : 1 alloys than the alloys with threefold ensembles is one of the primary reasons for the higher C–C bond breaking barriers observed, thereby inhibiting the hydrogenolysis reactions on the site isolated alloys. The modification of the C–C cleavage transition state ensemble also explains why site isolated alloys have high selectivity regardless of the intrinsic selectivity of the active metal. By changing the crystal structure through alloying, sites that lead to unselective pathways are removed, and instead these pathways must occur on sites with higher reaction barriers, regardless of the affinity of the active metal for such reactions.

To further probe the extent to which the structure of the metal ensembles, as opposed to the chemical identity of the promoter, is responsible for the large change in energetics in the 1 : 1 alloy systems, a theoretical Pd_3_Zn alloy with the AuCu_3_ crystal structure was simulated (Fig. S10†). This theoretical alloy contains the same four atom hydrogenolysis ensembles present in the other Pd_3_M alloys, and if the chemical identity of Zn is predominantly responsible for selectivity, then its binding energies and activation energy barriers for C–C and C–H bond cleavage would fall closer to those of the 1 : 1 PdZn alloys than those of the other 3 : 1 alloys. However, the calculated binding energies and activation energy barriers for the Pd_3_Zn structure (Table S8†) do indeed fall within the range of other Pd_3_M alloys simulated. This result suggests that the structure of the metal ensembles, rather than identity of the promoter, that is predominantly responsible for the high dehydrogenation selectivity observed for 1 : 1 alloys.

Finally, we note that the superior selectivity of 1 : 1 alloys, as compared to the 3 : 1 or 2 : 1 alloys, at high reaction temperatures, can also be influenced by the lower sensitivity of the 1 : 1 alloy structures to surface segregation of Pd atoms. All of the 3 : 1 alloy structures considered (and more generally most L1_2_ alloys) are stable over a narrow (2–5 at%) composition range, which is facilitated by partial occupancy of Pd at M lattice sites and *vice versa*. For example, this could allow metastable exchange of a surface M atoms with a subsurface Pd atom on a Pd_3_M surface, leading to fourfold Pd ensembles with lower olefin selectivity. Formation of such a defect through surface pre-melting in nanoparticles at high temperature is well documented.^70^ While the same partial occupancy can occur for similar reasons in 1 : 1 alloys, the occurrence of fourfold Pd ensembles by elemental exchange is less likely because multiple atom exchanges would need to occur in close proximity to produce atomic ensembles with maximum propensity for hydrogenolysis. The initial segregation analysis performed using DFT further illustrates this point (Table S9†); the 1 : 1 alloys have higher thermodynamic barriers for undergoing metastable exchanges of surface M atoms with sub-surface Pd atoms, as compared to 3 : 1 alloys, further leading to their superior selectivity.

## Conclusions

Simple synthetic methods, such as strong electrostatic adsorption and incipient wetness impregnation, allowed for the synthesis of 1–2 nm intermetallic compounds between Pd and five different promoters: Zn, Ga, In, Fe and Mn. All the alloys had higher dehydrogenation turnover rates and selectivities than those of an equivalently sized monometallic Pd catalyst. The site isolated alloys, PdIn and PdZn, had the highest selectivity among the alloys due to an order of magnitude decrease in the rate of hydrogenolysis as compared to alloys without site isolation. The change in selectivity and turnover rate in all the alloys resulted from the heteroatomic bonds between Pd and the promoter metal atoms, which led to electronic modification of Pd and changes in the crystal structure of the alloy. Computed selectivity descriptors involving the energy difference between propylene desorption and propylene dehydrogenation qualitatively match the experimental trends that site isolated Pd alloys have higher selectivity than Pd_3_M alloys. Modeling the C–C bond cleavage of propynyl, a representative C_3_ species resulting from deep dehydrogenation of propylene, showed a difference in the number of surface atoms involved in the hydrogenolysis reaction in the 1 : 1 alloys and in the alloys containing threefold ensembles, with implications for the design of new selective dehydrogenation catalysts. The relation of the selectivity descriptor to the reaction mechanism implies that it can be extended to other catalysts having the same dehydrogenation and hydrogenolysis mechanisms. This further implies that such an approach could be fruitful for other systems, such as base metal alloys and metal phosphides.

## Conflicts of interest

There are no conflicts to declare.

## Supplementary Material

SC-011-D0SC00875C-s001
