# Supplementary material for: Structural trends in the dehydrogenation selectivity of palladium alloys
Source: Chem Sci. 2020 May 5;11(19):5066–81. doi: 10.1039/d0sc00875c (PMC8159209; doi:10.1039/d0sc00875c)
Supplement: SC-011-D0SC00875C-s001 [file SC-011-D0SC00875C-s001.pdf]

Electronic Supplementary Material (ESI) for Chemical Science.
This journal is © The Royal Society of Chemistry 2020

Supplemental information:

Structural Trends in the Dehydrogenation Selectivity of Palladium Alloys

Stephen C. Purdy[1‡], Ranga Rohit Seemakurthi[1‡], Garrett M. Mitchell[1], Mark Davidson[1], Brooke Lauderback[1], Siddharth Deshpande[1], Zhenwei Wu[1], Evan Wegener[1], Jeffrey P. Greeley[1], Jeffrey T. Miller[1]

[1]Davidson School of Chemical Engineering, Purdue University, West Lafayette, IN 47907, USA

Keywords: palladium, nanoparticle alloy catalyst, propane dehydrogenation, in-situ synchrotron X-ray diffraction, in-situ X-ray absorption spectroscopy,

Table of Contents

Supplemental methods 3

Catalyst Synthesis 3

Pd 3

Pd-In 3

Pd-Fe 4

Pd-Ga 4

Pd-Zn 5

Pd-Mn 5

Pd L_3 edge XANES 6

Supplemental results 6

Electronic Characterization 10

Electronic Structure Calculations 12

Adsorption energies of deep dehydrogenated intermediates 13

Effect of vdW functionals on Pd (111) and PdIn (110) 14

Most stable adsorption configurations for reaction intermediates 17

Transition states for C-H and C-C bond breaking 18

Theoretical Pd_3Zn alloy results 19

Surface Segregation Energies 19

Supplemental discussion 20

References 21

Supplemental methods

Catalyst Synthesis

Pd

A Monometallic 2% Pd catalyst was prepared by strong electrostatic adsorption (SEA). 5 grams of davasil 646 silica was dispersed in 50 mL of deionized (DI) water and the pH was adjusted to 11 with 32% ammonium hydroxide. 2.8g of 10% palladium (II) tetraammine nitrate solution was diluted to a total volume of 25 mL and the pH was adjusted to 11 by the addition of 32% ammonium hydroxide. The silica suspension and the palladium solution were mixed for 15 minutes. The silica was then allowed to settle out of solution and removed by filtration. The Pd-SiO_2 was then washed 3 times with DI water and dried at room temperature for 3 hours and then overnight at 125°C. After drying the catalyst was calcined at 300°C for 3 hours. The Pd-SiO_2 was then reduced in 5% H_2 in steps from 100°C to 250°C at a 2.5°C/min ramp rate with 15 minute dwells every 25°C and then a fast ramp (10 C/min) to 550°C and a 30 minute dwell. The catalyst was then cooled to room temperature and passivated in air. A second monometallic Pd catalyst was synthesized by the above method except the calcination temperature was 200°C for 3 hours and the mass of 10% palladium (II) tetraammine nitrate was adjusted to give a weight loading of 1%.

Pd-In

A 2% Pd 3% In catalysts was synthesized according to the procedure for Pd-In 0.8 in reference[28]. Briefly, In-SiO_2 was synthesized by incipient wetness impregnation of an $In(NO_3)_2$ and Citric acid solution pH adjusted to 11 with ammonium hydroxide. The In-SiO_2 was dried at 125°C overnight and then calcined in air at 200°C. Palladium was then added by incipient wetness impregnation of a pH 11 solution of palladium (II) tetraammine nitrate. The Pd-In-SiO2 catalyst was then dried

overnight at 125°C and calcined at 200°C. The catalyst was then reduced using a slow ramp to 200°C and then a fast ramp to 600°C in 5% H_2 (balance N_2). The catalyst was cooled to room temperature in nitrogen and then passivated in air.

<u>Pd-Fe</u>

A 2% Pd 3%Fe catalyst was synthesized according to the procedure for "Pd_3Fe small" in reference[29]. A 2:1 molar ratio of citric acid to iron (III) nitrate nonahydrate was pH adjusted to 11 using concentrated ammonium hydroxide. The solution was impregnated dropwise onto 5g of SiO_2 and dried at 125°C and then calcined at 400°C for 3 hours. Pd loading was accomplished using a pH 11 solution of palladium (II) tetraammine nitrate. The Pd-Fe-SiO_2 catalyst was then dried at 125°C and then calcined at 250°C for 3 hours. Reduction was performed in 3% H_2 (balance Ar) at 200°C for 30 minutes and then at 600°C for 30 minutes. The catalyst was then cooled to room temperature and passivated in air.

<u>Pd-Ga</u>

A 2.5% Pd 2.5% Ga catalysts was prepared by sequential incipient wetness impregnation of gallium and palladium on silica. 1.25 g of gallium (III) nitrate hydrate and 2 grams of citric acid were dissolved in DI water to a total volume of 5 mL. The pH was adjusted to 11 with 32% ammonia solution and subsequently impregnated into 5 g of davasil 646 silica. The Ga-SiO_2 was dried at 125°C overnight and then calcined at 400°C for 3 hours. Pd impregnation was done using 3.3 g of 10% palladium (II) tetraammine nitrate diluted to 5 mL total volume and pH adjusted to 10 with 32% ammonia solution. The Pd solution was then impregnated to the pore volume of the 5 g of Ga-SiO_2 catalyst and then dried at 125°C overnight. The Pd-Ga catalyst was then calcined at 250°C for 3 hours and then subsequently reduced in 5% H_2 with a slow ramp (2.5°C/min)

through 200°C and then a fast ramp (10°C/min) to 600°C with a 30 minute dwell. The reduced catalyst was then cooled to room temperature in 5% H_2 and passivated in air.

Pd-Zn

A 2% Pd 3% Zn catalyst was synthesized by sequential incipient wetness impregnation. 1.14 g of Zinc nitrate hexahydrate was dissolved in 2 mL of DI water and the pH was adjusted to 11 using 32% ammonia solution. Finally, the total volume of the Zn solution adjusted to 5 mL with the addition of DI water. The Zn solution was impregnated to the pore volume of 5 g of davasil 646 silica and dried overnight at 125 C. The Zn-SiO_2 catalyst was then calcined at 300°C for 3 hours. 0.281 g of palladium (II) tetraammine nitrate was dissolved in 3.5 mL of DI water and the pH was adjusted to 11 using 32% ammonia solution. The palladium solution was then impregnated to the pore volume of the calcined Zn-SiO_2 and dried at 125°C. The Pd-Zn-SiO_2 catalyst was calcined at 200°C for 3 hours and then reduced in 5% H_2 (balance N_2) with a slow ramp (2.5°C/min) through 250°C and a fast ramp (10°C/min) to 550°C and a 30 minute dwell at temperature. The reduced catalyst was then cooled to room temperature in hydrogen and passivated in air.

Pd-Mn

A 1% Pd 5% Mn catalyst was synthesized by sequential incipient wetness impregnation. 0.814 g of manganese (II) nitrate hydrate and 0.874 g of citric acid were dissolved in 5 mL of Millipore water to give a solution with a 2:1 molar ratio of citric acid to manganese nitrate. The pH of the solution was adjusted to 11 by the addition of 32% ammonium hydroxide. The solution was then added dropwise to 5 g of davasil 646 silica. The Mn-SiO_2 was then dried at 125°C overnight and calcined at 250°C for 3 hours. Pd loading was done by diluting 1.4 g of 10% palladium (II) tetraammine nitrate to 5 mL total volume and adjusting the pH to 11 with 32% ammonium hydroxide. The solution was then added dropwise to the Mn-SiO_2 and dried at 125°C overnight

and calcined at 200°C for 3 hours. The Pd-Mn-SiO_2 catalyst was then reduced in 5% H_2 (balance N_2) with a slow ramp (2.5°C/min) through 250°C and a fast ramp (10°C/min) to 550°C with a 30-minute dwell at temperature. The reduced catalyst was then cooled to room temperature in 5% H_2 and passivated in air.

Pd L_3 edge XANES

Pd L_3 edge X-ray adsorption near edge structure (XANES) were measured at the 9BM line of the advanced photon source. Measurements were performed in fluorescence mode using a vortex 4 element detector. The samples were ground and pressed into a steel sample holder with the catalyst wafer at a 45-degree angle relative to the beam. The reactor used for treatment has been described elsewhere[1], and is capable of heating and gas flow with kapton windows for transmission and fluorescence measurements. Samples were treated by heating to 500°C in 3.5% H_2 (balance He). After a 30-minute dwell at 500°C, the gas flow was switched to He at high temperature to desorb hydrogen and decompose any palladium hydride formed during the reduction. The samples were then cooled to room temperature and multiple scans were collected and averaged. L_3 edge XANES spectra were normalized using first order polynomial for the pre-edge region and a second order polynomial for the post-edge region. Due to the close proximity of the L_3 to the L_2 edge, the data collection range for post edge normalization is limited and the third order polynomial typically fit to the post edge region fits poorly. The absolute energy scale was calibrated using bulk PdO with an L_3 edge energy of 3174.4 eV.

Supplemental results

Figure S1 shows STEM images and EDX maps of Pd and Pd alloy catalysts. Pictured in figure S1a, the 1% Pd catalyst is monodisperse with small metal particles under 2 nm in diameter. In

contrast, the 2% Pd catalyst, shown in figure S1b, contains both small (1-2 nm) particles and large (5+ nm) particles. The high temperature calcination treatment used in the 2Pd catalyst results in agglomeration of the palladium oxide resulting in larger metallic particles after reduction. EDX was used to observe the dispersion of the second metal, which is difficult to distinguish from the support in STEM images. Figure S1c shows a STEM image of 1Pd-5Mn with overlaid EDX maps for manganese and palladium. The manganese is well dispersed across the support, small clusters containing both Pd and Mn can be seen, consistent with the formation of a Pd-Mn bimetallic. Ga in the Pd-Ga catalyst (figure S1d) is also well dispersed on the support, but the bimetallic Pd-Ga clusters are better resolved owing to their slightly larger particle size.

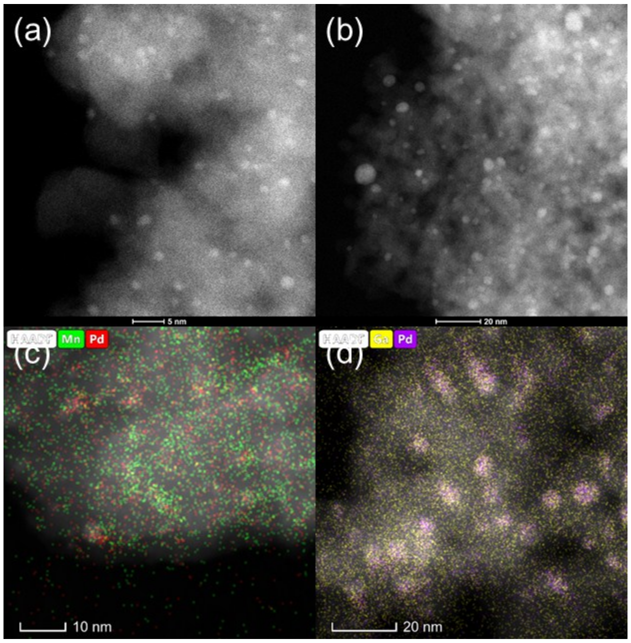

Figure S1: STEM images of Pd and bimetallic Pd catalysts: (a) 1Pd (b) 2Pd (c) STEM image of 1Pd-5Mn with overlaid EDX maps for Pd (red) and Ga (Green). (d) STEM image of 2.5Pd-2.5Ga with overlaid EDX maps for Pd (purple) and Ga (yellow)

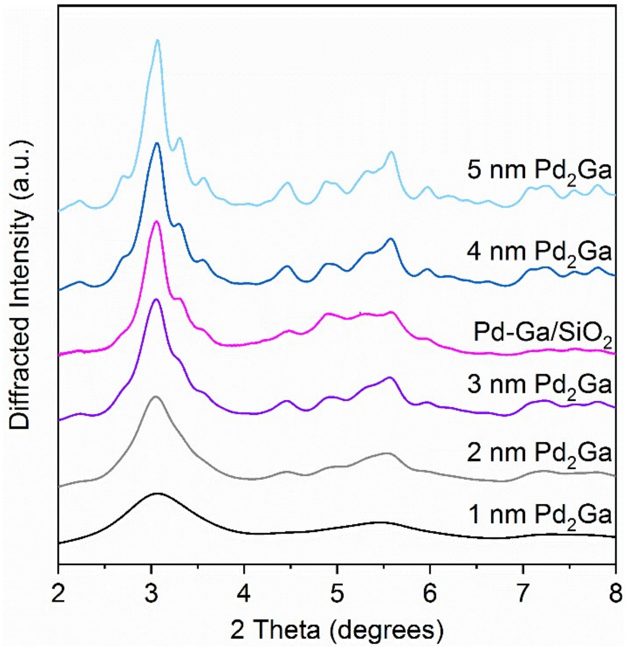

Figure S2: Simulated Pd_2Ga XRD patterns with different crystallite sizes (black, grey, purple, blue, light blue) compared with experimentally measured Pd-Ga catalyst (pink).

Due to the large number of overlapping peaks and imperfect background subtraction, it was not possible to determine a lattice parameter using the above pattern. However, simulation of the particle size broadening, shown in figure S5, gives an estimate of the particle size. The first major group of peaks, between 2.5-4 degrees, merges into a single asymmetric peak when the particle size is below 2 nm. In the 3 and 4 nm sized simulations, distinct shoulders start to emerge, and 4 major peaks can be resolved. In the 5 nm simulation, the most intense peak in the pattern around 3.1 degrees starts to split into two distinct peaks. In the Pd-Ga catalyst, the first cluster of peaks between 2.5-4 degrees most closely resembles the 3 nm simulation, which is larger the TEM measured particle size of 2.1. The difference between the XRD determined value and the TEM

determined value can be attributed to the presence of microstrain broadening and the volume averaging nature of the XRD measurement.

Table S1: Pd K edge EXAFS fits of Pd and Pd alloy catalysts after room temperature air exposure

Sample	Scattering Pair	Coordination number	Bond Distance (Å)	Debye-Waller factor (Å^2)	E_0 (eV)
1Pd	Pd-Pd	5.6	2.71	0.006	-3.2
	Pd-O	1.4	2.02	0.002	0.4
2Pd	Pd-Pd	8.6	2.74	0.0018	-2.0
	Pd-O	0.6	2.01	0.002	1.6
2Pd-3Zn	Pd-Pd	1.3	2.7	0.003	-3.6
	Pd-Zn	2.2	2.52	0.003	-9.6
	Pd-O	0.5	2.05	0.002	2.1
2Pd-3In	Pd-M	4.9	2.7	0.006	-3.3
	Pd-O	0.6	2.04	0.002	0.4
2.5Pd-2.5Ga	Pd-Pd	3.6	2.75	0.003	-1.8
	Pd-Ga	1.4	2.46	0.003	-9.2
	Pd-O	0.3	2.01	0.001	1.9
2Pd-3Fe	Pd-Pd	5.2	2.72	0.004	-0.2
	Pd-Fe	1.6	2.6	0.004	-4.4
	Pd-O	0.4	2.02	0.002	-1.6

Electronic Characterization

In addition to changing the catalyst structure, alloying can also electronically modify palladium, which can be studied by XANES. Figure S3a shows the Pd K edge XANES collected for 2Pd-3Zn,

2.5Pd-2.5Ga, 1Pd-5Mn and 1Pd after reduction in 3.5% H_2 (balance He) for 30 minutes. The alloy catalysts show small changes in the edge shape which are indicative of alloying. The first peak in the XANES is lower in intensity for the three alloy samples and the edge position for each is shifted 0.2 eV lower in energy compared to the Pd foil value of 24350.0 eV. The close edge position and white line intensity shows that the alloy sample and Pd nanoparticles are all in the metallic state. Because the final state in K edge absorption is a p electron, and transition metals bond and adsorb through d electron interactions, only small changes are evident at the Pd K edge. Larger modifications can be seen at the L_3 edge, which is more sensitive to adsorbates and heteroatomic bonding due to the final state involving unfilled valence s and d states.

Figure S3b shows the Pd L_3 edge XANES for 2Pd-3Zn, 2.5Pd-2.5Ga, 1Pd-5Mn and 1Pd after reduction at 500C in 3.5% H_2. The modification of the XANES due to alloying is evident in the intensity and broadness of the white line, demonstrating the sensitivity of the L_3 edge to heteroatomic bonding. The PdZn and PdGa catalysts look similar, both with white line intensities lower than and broader than Pd sample. The PdGa sample white line is slightly broader and higher in intensity compared to the PdZn sample. Conversely, the PdMn sample has a white line that is narrower and lower in intensity compared to the Pd catalyst. The edge energy for the Pd and PdMn catalysts was 3173.6 eV. For PdGa and PdZn, the edge energy increased to 3173.9 eV. The dipole selection rule for Pd L_3 edge XANES allows for transition of a $2p_{3/2}$ electron into an unfilled 4s or 4d state. Since metallic Pd is d_{10}, there are no unfilled d states and the changes in the Pd L_3 XANES reflect the 4s unfilled density of states (conduction band) which are just above the 4d electrons in energy. The change in white line shape for the alloys represents a redistribution of the energy of the unfilled s states resulting from alloy formation.

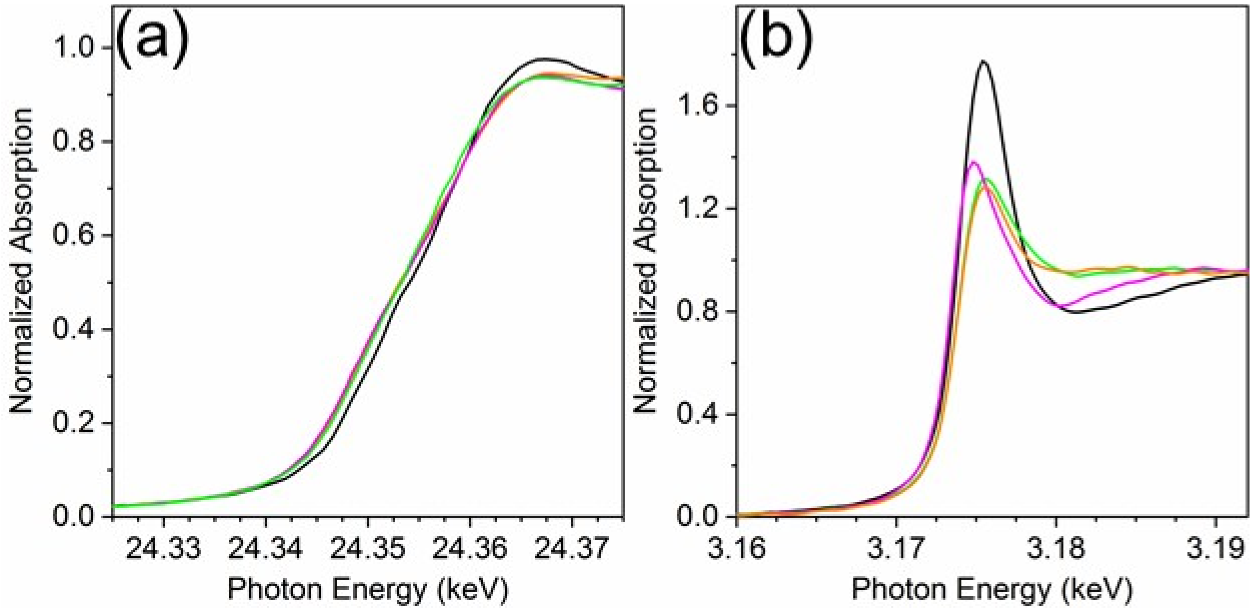

Figure S3: XANES of 1Pd (black), 2Pd-3Zn (orange), 2.5Pd-2.5Ga (green) and 1Pd-5Mn (magenta) at the K edge (a) and L_3 edge (b) after reduction at 550°C in 3.5% H2.

Electronic Structure Calculations

The surface Pd-atom projected Density of States (DoS) have been calculated for all the alloy surfaces to quantify the electronic modification of palladium in the alloy structures. Further, the 1st moment (d-band center) and 2nd moment (d-band width) are reported in Table S2. Apart from the Pd_2Ga surface, all the Pd atoms on the surface of the alloys are crystallographically identical. Pd_2Ga has two symmetrically distinct Pd atoms, hence an average of their moments has been reported.

Table S2: The d-band center (first moment) and d-band width (second moment) calculated from the pDOS of surface Pd atoms in monometallic Pd and Pd alloys

Structure	d-band center (first moment) (eV)	d-band width (second moment) (eV)
Pd (111)	-1.54	2.19

Pd_3Mn/Pd (111)	-1.67	2.30
Pd_3Fe (111)	-1.85	2.51
Pd_2Ga (010)	-1.95	2.55
PdZn (101)	-2.07	2.70
PdIn (110)	-2.16	2.66

The results show that as the promoter content increases the Pd d-band center shifts away from the fermi level leading to more negative d-band centers. The decrease in d-band center is also accompanied by a concomitant increase in d-band width. This inverse linear correlation has also been observed for Pt-skin alloys by Nikolla et al[2]. The changes are smallest for Pd_3Mn/Pd, which also showed the smallest changes in binding strengths. The alloys that had the largest binding energy shifts (PdIn and PdZn) also showed the largest movement in the d-band center, in agreement with the d-band theory. Both Pd and the Pd alloys had a negligible amount unfilled d states above the Fermi level which is consistent with palladium being d^{10} in all cases and the L_3 edge XANES modifications coming from s unfilled state modification.

Adsorption energies of deep dehydrogenated intermediates

Table S3: Binding energies of deep dehydrogenated intermediates with respect to gas phase reference

Adsorbate	Pd (111)	Pd_3Mn/Pd (111)	Pd_3Fe (111)	Pd_2Ga (010)	PdZn (101)	PdIn (110)
Propyne	1.75	2.02	2.18	2.54	3.13	3.59
Propynyl	2.72	2.78	2.41	3.01	3.60	3.96
Methylidyne	1.30	1.67	1.86	2.19	3.20	3.13
Carbon	2.15	2.20	2.31	3.07	4.47	4.29

Table S4: Binding energies of deep dehydrogenated intermediates on Fe (110) and their difference from Pd (111)

Fe (110)	Binding Energy (eV)	Difference from Pd (111)
Propyne	0.69	-1.06
Propynyl	1.40	-1.32
Methylidyne	0.72	-0.58
Carbon	1.22	-0.93
Ethylidyne	0.58	-0.38

Table S5: Activation energy barriers for C-C bond cleavage of propyne on Pd and Pd alloy surfaces

Alloy surfaces	Kinetic Barrier (eV)
Pd (111)	1.1
Pd_3Mn/Pd (111)	1.5
Pd_3Fe (111)	0.92
Pd_2Ga (010)	1.84
PdZn (101)	2.55
PdIn (110)	2.58

Effect of vdW functionals on Pd (111) and PdIn (110)

To understand whether the functional influences the selectivity trends obtained, the binding energies of all the C_3 intermediates on Pd (111) and PdIn (110) have been recalculated with BEEF-vdW and optPBE functionals (Tables S6 and S7). The two surfaces have been chosen as

representing the two extremes in terms of binding of adsorbates. The results show that the binding of intermediates are stronger with the vdW functionals, with optPBE having the strongest binding among the three functionals. Interestingly, on both the surfaces, the binding energies also have a linear correlation with the values obtained using PBE functionals (Figures S4 and S5). The slopes of the correlations are approximately 1, with the intercept being more negative for optPBE than for BEEF-vdW. This illustrates that the vdW functionals increase the strength of binding of intermediates by a constant value, given by the intercept. Therefore, the changes in reaction energetics and barriers for dehydrogenation on the surfaces would be very small. We expect these linear correlations to hold for other alloy surfaces as well. Furthermore, the calculated differences in binding energies of propylene, between Pd (111) and PdIn (110), are 0.71 eV for PBE, 0.67 eV for BEEF-vdW, and 0.83 eV for optPBE. The differences between the functionals are within 0.15 eV of each other, thereby preserving the selectivity trends obtained using the PBE functional.

Table S6: Adsorption energies of C_3 reaction intermediates on Pd (111) using PBE, BEEF-vdW and optPBE functionals

Species	PBE	BEEF-vdW	optPBE
propylene	-0.77	-0.95	-1.27
1-propenyl	1.55	1.34	1.02
2-propenyl	1.48	1.28	0.95
propyne	1.81	1.56	1.28
propynyl	2.72	2.36	2.17

Table S7: Adsorption energies of C_3 reaction intermediates on PdIn (110) using PBE, BEEF-vdW and optPBE functionals

Species	PBE	BEEF-vdW	optPBE
propylene	-0.06	-0.28	-0.44
1-propenyl	2.70	2.48	2.20
2-propenyl	2.80	2.56	2.27
propyne	3.59	3.41	3.04
propynyl	3.93	3.61	3.32

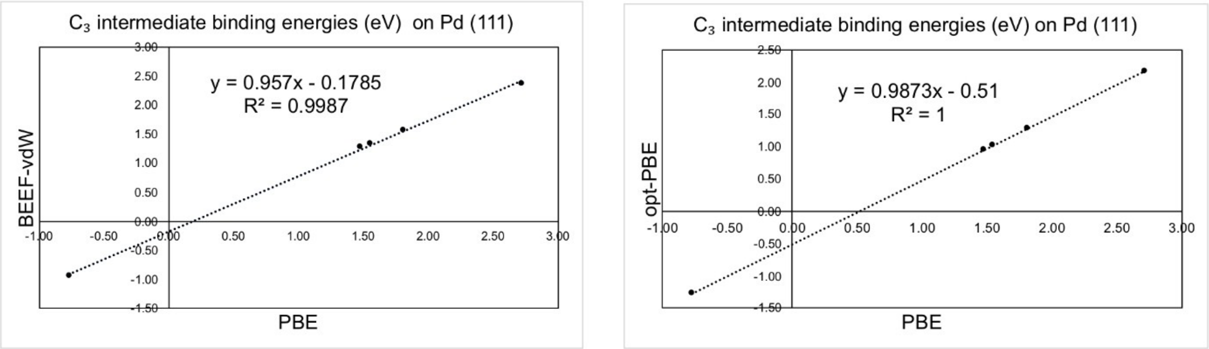

Figure S4: Plot of binding energies of C_3 intermediates with different functionals (a) BEEF-vdW vs PBE (b) optPBE vs PBE

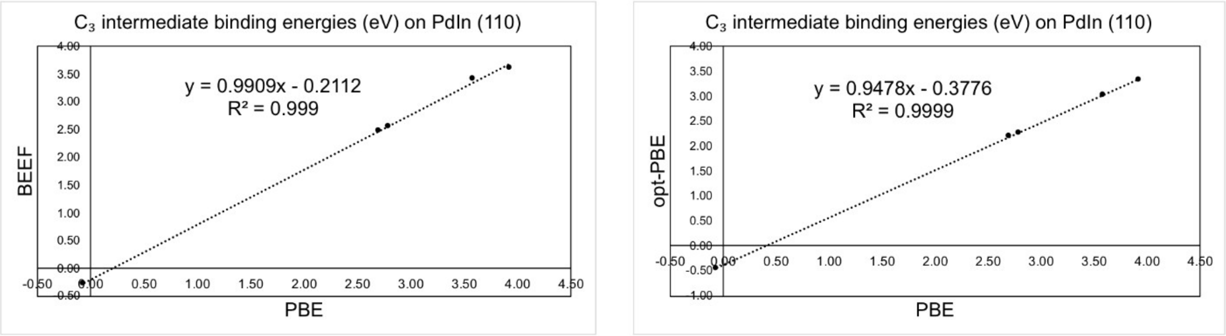

Figure S5: Plot of binding energies of C_3 intermediates with different functionals (a) BEEF-vdW vs PBE (b) optPBE vs PBE

Most stable adsorption configurations for reaction intermediates

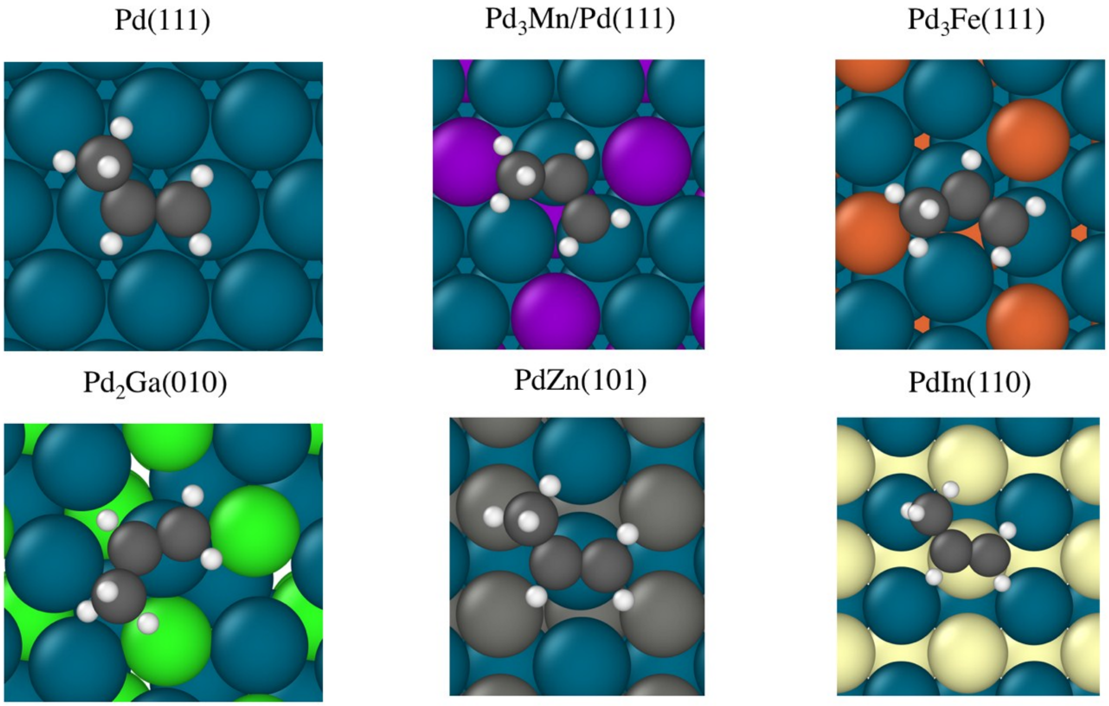

Figure S6: Propylene most stable configurations on alloy and pure Pd surfaces

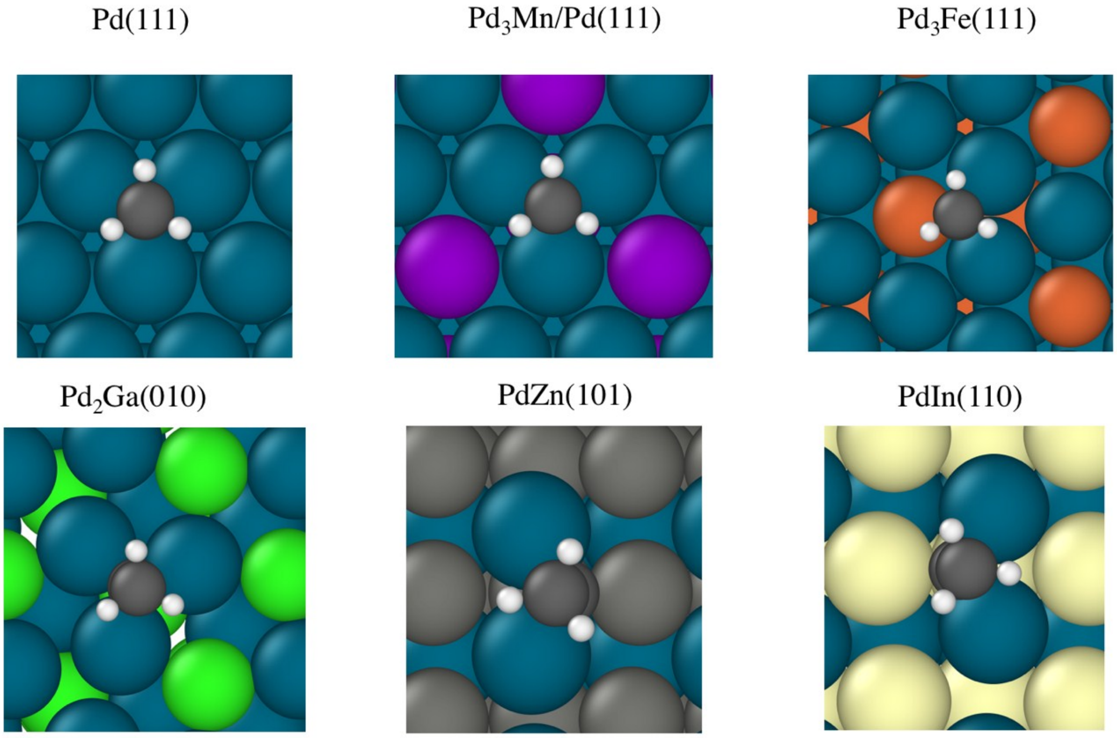

Figure S7: Ethylidyne most stable configurations on alloy and pure Pd surface

Transition states for C-H and C-C bond breaking

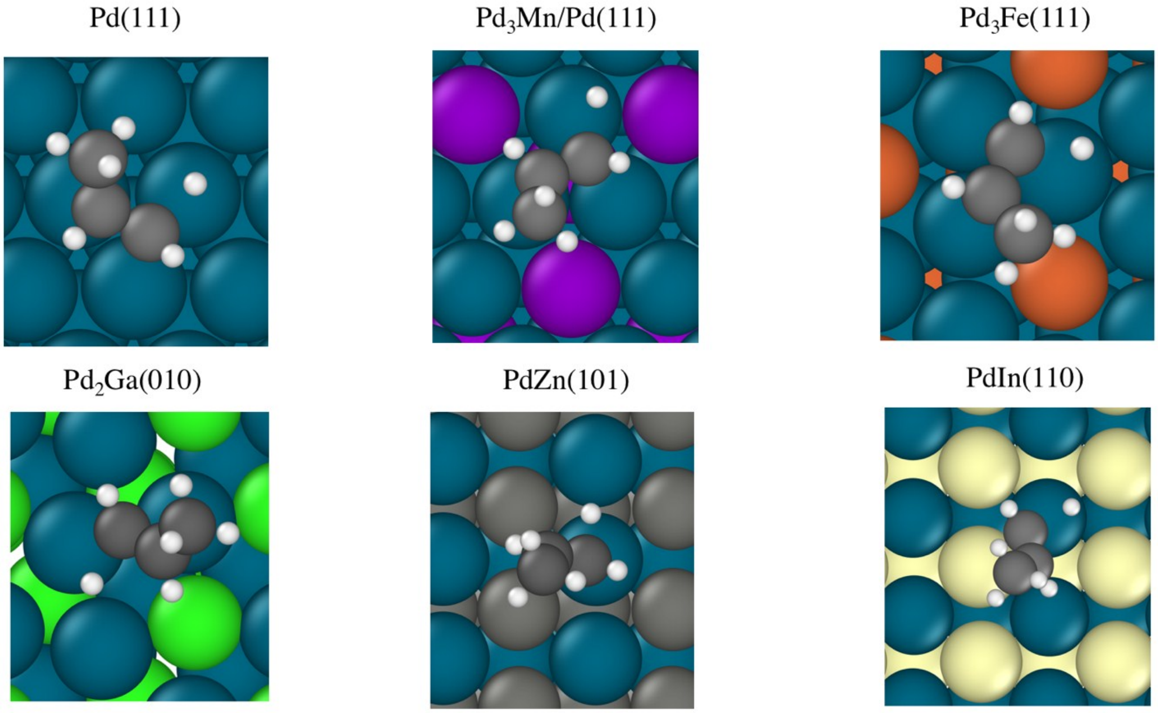

Figure S8: Tranisiton states of propylene C-H bond breaking to form propenyl and hydrogen

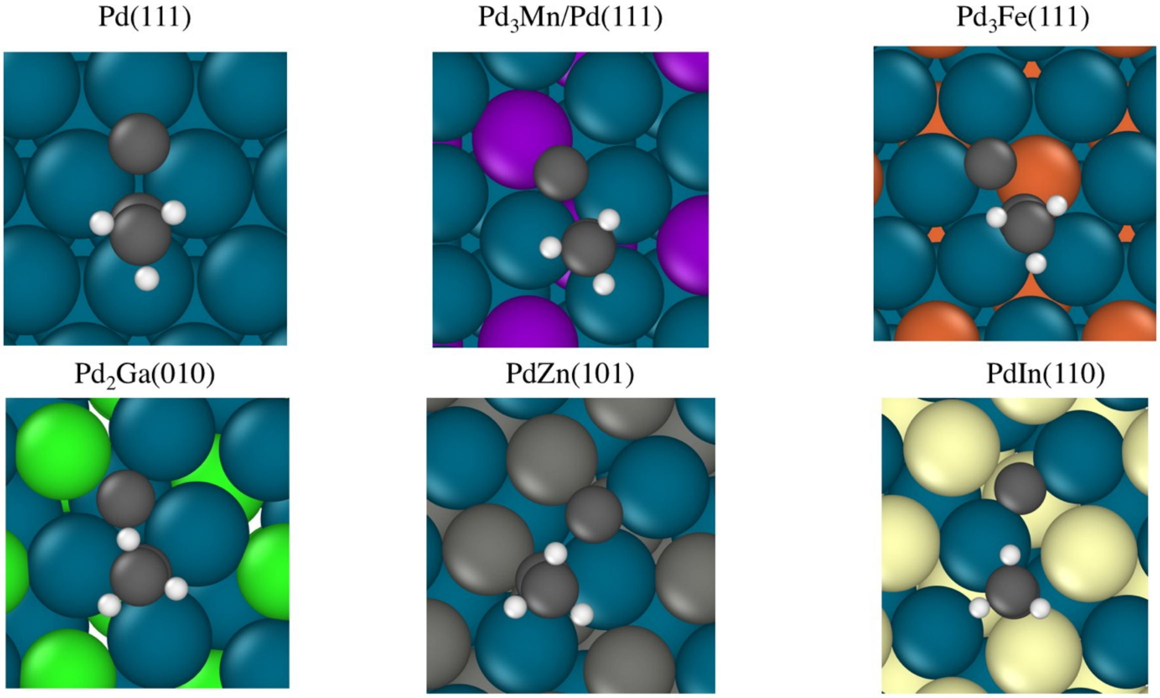

Figure S9: Transition states of propynyl C-C bond breaking to form carbon and ethylidyne

Theoretical Pd_3Zn alloy results

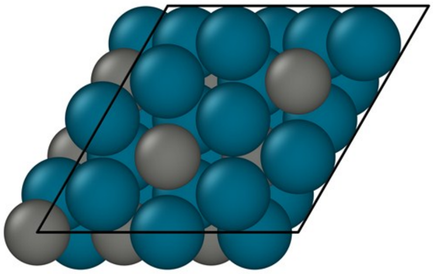

Figure S10: Top view of fcc Pd_3Zn (111) surface

Table S8: Calculated values of selectivity descriptor and propynyl C-C bond breaking barriers for Pd_3Zn (111) surface

Alloy surface	Propylene Adsorption Energy (eV)	Propylene dehydrogenation barrier (eV)	Selectivity Descriptor (eV)	Propynyl C-C bond breaking barrier (eV)
Pd_3Zn (111)	-0.60	1.28	-0.68	1.98

Surface Segregation Energies

To analyze the propensity of the alloy terrace surfaces to form clusters of either Pd or promoter on the top layer of the surface, we have performed a simple thermodynamic analysis by swapping the atoms on the surface with a counter-atom from 2nd layer. This led to formation of surfaces with either a Pd-rich or a promoter rich top layer. The energies reported in Table S7 are the segregated energies of each alloy surface with respective to its clean surface counterpart.

Table S9: Segregation energies of surfaces with Pd-rich and promoter-rich top-layers with respect to its clean surface

Segregation Energy	Pd_rich (eV)	X_rich (eV)
Pd_3Mn/Pd (111)	-0.16	0.87
Pd_3Fe (111)	-0.34	0.63
Pd_2Ga (010)	1.02	1.49
PdZn (101)	1.49	1.13
PdIn (110)	1.94	0.74

The analysis demonstrates that for the 1:1 and 2:1 alloys, the tendency to form segregated surfaces is very low, considering their large thermodynamic barriers (> 1 eV). For the 3:1 alloys, even though the energetics to form promoter-rich surfaces are still above 0.5 eV, there exist slight driving forces to form Pd-rich surfaces. Although we would not expect such small values to lead to substantial compositional changes in the surface layers, this effect could modestly contribute to the lower selectivity of 3:1 alloys towards propylene formation in comparison to 1:1 alloys.

Supplemental discussion

Because the structure of the alloy determines the local Pd coordination in an intermetallic compound, it also determines the electronic effect, and the two effects cannot be decoupled. In addition to the difference in ensemble size between the alloys, they also differ in the number of surface Pd-Pd and Pd-promoter bonds. The Electronic effect occurs due changes in the local coordination sphere of the surface atoms, and the number of bonds changes the strength of the electronic modification. This has been demonstrated in the Pt-Mn system, where the subsurface alloy or Pt layer of the catalyst changed the CO heat of adsorption of the Pt_3Mn surface[3]. In the site isolated PdZn alloy (Figure 8), each surface Pd atom on the lowest energy surface has 6 heteroatomic bonds (4 surface, 2 subsurface) and 2 Pd second nearest neighbors at a non-bonding

distance (2.89 Å); PdIn has the same number of herteroatomic bonds but 6 Pd second nearest neighbors at 3.22 Å. Pd atoms on the (010) surface of Pd_2Ga have 3 Ga neighbors (2 surface, one subsurface) with 4 Pd nearest neighbors at an elongated bond distance (2.8, 2.85 Å). Lastly, surface atoms in the Pd_3M structures have 3 promoter nearest neighbors (2 surface, 1 subsurface) with 6 Pd-Pd bonds equal in length to the Pd-promoter bonds. As the local coordination changes, the d band of palladium is modified, which is quantified in the first and second moments of the d band. In general, as the number of Pd-promoter bonds increases, the d band shifts away from the fermi level and increases in width (see table S2).

While L_3 edge XANES is commonly used to demonstrate d-band modification in platinum, the same information cannot be gained at the Pd L_3 edge due to the electron configuration of Pd. Because Pd metal is d_{10}, there are no unfilled d states, and the L_3 edge XANES cannot give information about the d-band. The lowest energy unfilled state accessible by the dipole selection rules of XANES is the 5s unfilled states. The change in whiteline shape observed for the alloys reflects the unfilled s states redistributing in energy due to overlap with neighboring promoter s orbitals, similar to how the 5d unfilled states in platinum are modified by promoters in platinum alloys[4].

References

(1) Bolin, T. B.; Wu, T.; Schweitzer, N.; Lobo-Lapidus, R.; Kropf, A. J.; Wang, H.; Hu, Y.; Miller, J. T.; Heald, S. M. In Situ Intermediate-Energy X-Ray Catalysis Research at the Advanced Photon Source Beamline 9-BM. Catal. Today **2013**, 205, 141–147. https://doi.org/10.1016/j.cattod.2012.09.034.

(2) Nikolla, E.; Schwank, J.; Linic, S. Measuring and Relating the Electronic Structures of Nonmodel Supported Catalytic Materials to Their Performance. J. Am. Chem. Soc. **2009**, 131 (7), 2747–2754. https://doi.org/10.1021/ja809291e.

(3) Wu, Z.; Bukowski, B. C.; Li, Z.; Milligan, C.; Zhou, L.; Ma, T.; Wu, Y.; Ren, Y.; Ribeiro, F. H.; Delgass, W. N.; et al. Changes in Catalytic and Adsorptive Properties of 2 Nm Pt3Mn Nanoparticles by Subsurface Atoms. J. Am. Chem. Soc. **2018**. https://doi.org/10.1021/jacs.8b08162.

(4) Schweitzer, N.; Xin, H.; Nikolla, E.; Miller, J. T.; Linic, S. Establishing Relationships Between the Geometric Structure and Chemical Reactivity of Alloy Catalysts Based on Their Measured Electronic Structure. Top. Catal. **2010**, 53 (5–6), 348–356. https://doi.org/10.1007/s11244-010-9448-1.
